# Analysis and Interpretation of the Impact of Missense Variants in Cancer

**DOI:** 10.3390/ijms22115416

**Published:** 2021-05-21

**Authors:** Maria Petrosino, Leonore Novak, Alessandra Pasquo, Roberta Chiaraluce, Paola Turina, Emidio Capriotti, Valerio Consalvi

**Affiliations:** 1 Dipartimento Scienze Biochimiche “A. Rossi Fanelli”, Sapienza University of Rome, 00185 Roma, Italy; maria.petrosino@uniroma1.it (M.P.); leonore.novak@uniroma1.it (L.N.); roberta.chiaraluce@uniroma1.it (R.C.); 2 ENEA CR Frascati, Diagnostics and Metrology Laboratory FSN-TECFIS-DIM, 00044 Frascati, Italy; alessandra.pasquo@enea.it; 3 Dipartimento di Farmacia e Biotecnologie (FaBiT), University of Bologna, 40126 Bologna, Italy; paola.turina@unibo.it

**Keywords:** protein structure, protein stability, protein function, single amino acid variant, putative cancer driving variant, free-energy change

## Abstract

Large scale genome sequencing allowed the identification of a massive number of genetic variations, whose impact on human health is still unknown. In this review we analyze, by an in silico-based strategy, the impact of missense variants on cancer-related genes, whose effect on protein stability and function was experimentally determined. We collected a set of 164 variants from 11 proteins to analyze the impact of missense mutations at structural and functional levels, and to assess the performance of state-of-the-art methods (FoldX and Meta-SNP) for predicting protein stability change and pathogenicity. The result of our analysis shows that a combination of experimental data on protein stability and in silico pathogenicity predictions allowed the identification of a subset of variants with a high probability of having a deleterious phenotypic effect, as confirmed by the significant enrichment of the subset in variants annotated in the COSMIC database as putative cancer-driving variants. Our analysis suggests that the integration of experimental and computational approaches may contribute to evaluate the risk for complex disorders and develop more effective treatment strategies.

## 1. Introduction

**Figure 1:**
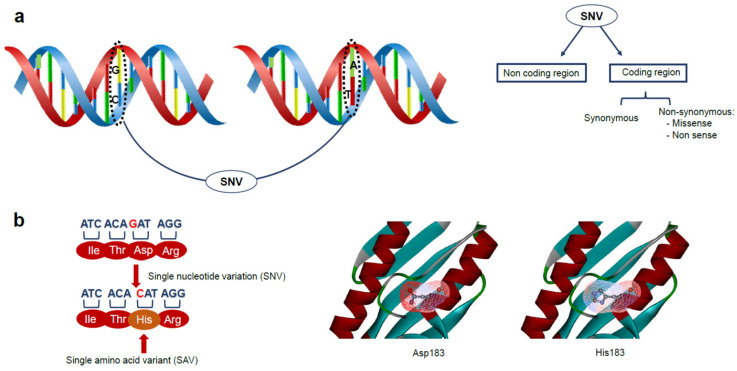
(**a**) Single nucleotide variations (SNVs) can occur in the coding or in the non-coding region. SNVs in the coding region can be synonymous if no amino acid changes are produced, non-synonymous if the single nucleotide substitution induces changes in the protein sequence. Usually, two types of non-synonymous changes can be described: missense mutation, that produces an amino acid change in the protein (SAV) and nonsense mutation which produces a truncated or a longer protein. (**b**) A single nucleotide substitution can lead to a single amino acid change generating a protein variant with structural and/or functional alterations as shown in the substitution of the residue Asp183 with a His in the human frataxin protein (PDB code: 1EKG).

Recent advances in the high-throughput sequencing technologies have led to the detection of a large amount of genomic data, which are used for generating detailed catalogues of genetic variations in both diseased and healthy patients [1,2]. Such genetic differences are at the basis of distinctive traits associated with the susceptibility to specific disease and/or drug response [3]. The most common genetic differences in the human genome are single nucleotide polymorphisms (SNPs), which are defined as single nucleotide variations (SNVs) occurring with a frequency of more than 1% in the population [4,5]. These differences occur on average once every 300–400 base pairs [6], either in coding or in non-coding regions (Figure 1). SNVs may affect exon splicing or transcription [3], and are found more frequently than other types of genetic variations, such as differences in copy number, insertions, deletions, duplications, and rearrangements. SNVs in protein-coding regions have received the most attention, in spite of the fact that those regions account for only about 2% of the total human genome [7]. SNVs in the coding region can be synonymous if no amino acid change is produced, or non-synonymous if the substitution leads to a change in the protein sequence. The non-synonymous variants can be further divided into two categories: missense mutations, which lead to single amino acid changes, or nonsense mutations, which produce truncated or longer proteins (Figure 1).

Missense mutations, that generate protein variants with a single amino acid variation (SAV), are of particular interest in biomedicine, since even just a single amino acid substitution may induce drastic structural alterations, which compromise the protein stability, or may induce crucial structural alterations able to perturb binding interfaces, to the point of impairing the protein function [8,9] (Figure 1).

The huge numbers of variants identified over the past twenty years have been collected in several databases, the most representative of which are summarized in Table 1. These databases represent the main source of information for studying the effect of protein variants and understanding the genotype/phenotype relationship [10].

To maximize the impact of sequence technologies in clinical settings, the scientific community is defining standard protocols and guidelines for discriminating disease-causing variants from nonpathogenic ones [11]. As a consequence, a large variety of genetic alterations, including nonsynonymous single nucleotide variants (nsSNVs), were found to be associated with monogenic and multigenic diseases [12,13], such as type II diabetes mellitus [14], acute lymphoblastic leukemia [15], or cancer [16,17]. Nevertheless, for the vast majority of variants, their impact at phenotypic level is still unknown. In this framework, the structural and functional analysis of nsSNVs proteins by experimental approaches can significantly contribute to their phenotypic characterization [18]. In particular, it has been recognized that most disease-causing variants affect the protein thermodynamic stability, expressed as the difference in folding free energy between the native and the denatured state (ΔG_f_). The experimental determination of the difference in ΔG between the mutant and wild-type proteins (ΔΔG_f_) constitutes a first and essential analysis for the characterization of the protein variants.

For most of the pathogenic variants, a strongly destabilizing mutation corresponds to the loss of function, whereas a modest change in stability may generate changes in protein conformation affecting the binding affinity with interacting molecules (protein, RNA and DNA). However, the impact of amino acid substitution on protein stability is an essential information for enabling precision medicine [25]. On such experimental bases, computational tools, which predict mutation-driven ΔΔG values at high levels of performance [26], allow a fairly reliable estimate of the variant effects on the large genomic scale.

In this work, we analyzed a set of 164 cancer-related missense variants, for which the ΔΔGs were experimentally determined. On such a dataset, we applied computational methods for predicting the variants induced change in folding free energy [27], and the variants pathogenicity [28]. The performance of the predictors was then further assessed by considering the variants annotation reported in the Cancer Mutation Census database from COSMIC [19].

## 2. Genetic Variations and Disease: The Role of Protein Stability in Cancer

Origin, onset, and progression of the neoplastic disease are generally driven by the accumulation of random genetic changes in cells and tissues, which can then develop independence from normal physiological controls due to randomly accumulating mutations, which disrupt the ability to recognize or respond to host signals [29]. This mechanism, generally referred to as “somatic evolution”, is part of the transition from a normal into a cancer cell [29]. While normal cells in metazoans generally lack the ability to evolve, cancer cells compete with each other, under limited resources, assuming the capacity to proliferate when stimulated by foreign antigens to maximize their fitness [29,30]. The elevated turnover of cancer cells, undergoing selective environmental pressure, may contribute to generating the high number of mutations found in tumors. Cancer may indeed be considered an adaptive evolutionary process [31,32,33], and most works correspondingly focus on the study of somatic mutations [16,34]. As shown by Genome Wide Association Studies (GWAS), many of these cancer-associated genetic variants are not necessarily the cause of the disease, they simply exist into the general picture of the neoplastic disorder and may contribute or not to the evolution of the clinical course of the pathology [35].

The accumulation of mutations in somatic cells represents the most likely event, however, germline mutations are also present in cancer, and they can be associated with cancer susceptibility, which can be passed on to subsequent generations. Public databases (Table 1) report a prevalence of putative pathogenic somatic variants [36], with respect to germline mutations [37,38]. It is reported that about 4.3–17.5% of cancer patients are characterized by a genetic heritage of germline variants [39,40,41,42,43], especially in the BRCA1, BRCA2, PTEN, TP53, KRAS, and CDH1 genes. Far greater is the number of somatic variants identified in oncogenes, or in tumor suppressor genes, as well as in a multitude of other genes. In particular, it has been recently suggested that somatic mutations may drive late-onset cancers and germline mutations may contribute to early-onset cancers [44].

Within the huge number of somatic and germline variants present in different cancer types, which are reported in online databases, one of the main issues is to identify those which are drivers (i.e., pathogenic) as opposed to those which are passengers (i.e., non-pathogenic). In the COSMIC database, for instance, several criteria enter into such classification effort, among which the variant frequency of occurrence in genes classified as oncogenes, or as Tumor Suppressor Genes (TSG), or the variant annotation in the ClinVar database as ‘pathogenic’ or ‘likely pathogenic’ in cancer-related genes. In turn, genes classified in COSMIC as oncogenes or TSG are intensely under the loupe for characterizing their product’s multiple functions and roles within the cell. Alongside such types of evidence, many computational predicting tools on the pathogenicity of protein variants are available, which are based on the available experimental data. These data were obtained on a tiny subset of all existing proteins and variants thereof, because of the time-consuming nature of the entire experimental process, i.e., mutagenesis, protein expression and purification, structural and functional characterization, that will never allow an exhaustive experimental characterization for all the SAVs. Nevertheless, it is evident that expanding the collection of experimental data will significantly improve the performance levels of the existing predictors, as well as increase the potential to generate novel and more accurate methods.

In addition to the criteria provided in COSMIC, other features can concur to predicting the carcinogenicity potential of a missense variant. Among such features, protein destabilization is a general phenomenon to be considered in all types of disorders. A structural destabilization may trigger protein misfolding and degradation by the ubiquitin-proteasome system [45,46,47], leading to an insufficient cellular amount of the SAV, which can be the cause of disease [48,49,50,51]. For TSG proteins, it has been shown that destabilizing or site-specific loss-of-function (LoF) variants promote cancer onset and cell proliferation [52,53], while for oncogene proteins the molecular mechanisms describing the pathogenic effects of variants are still largely unknown [54]. The main hypothesis is based on the acquisition of new protein functions associated with specific mutations. The characterization of such mutations, referred to as gain-of-function (GoF), has proven to be much more complex compared to LoF variants. This may be due to the combination of several factors. With respect to LoF, the GoF variants result in sequence and structural changes which may have regulatory effects or alter the binding to other proteins, to DNA or RNA molecules, or to other ligands [55]. Although a detailed discussion on the characterization of GoF variants is out of the scope of this report, in the final part of this review we discussed the case of five p53 hotspot variants in the DNA binding domain, which a large body of experimental evidence indicates as GoF [56].

## 3. Experimental Analysis of Protein Variants in Cancer-Related Genes

Here we present an analysis of missense variants in cancer-related genes, selected from different databases (Supplementary File S1), for which a ΔΔG_f_ has been experimentally determined. The missense variants of our dataset affect tumor suppressor genes, such as BRCA1 [57] and TP53 [58], or proteins involved in the regulation of cell metabolism, such as phosphoglycerate kinase 1 (PGK1) and human frataxin (hFXN) [59] as well as proteins involved in the epigenetic regulation of gene transcription and master transcriptional factors, such as bromodomains (BRDs) [60], protein kinase PIM-1, and Protein tyrosine phosphatase ρ (PTPρ), whose dysregulation can influence different signaling pathways [61], and peroxisome proliferator receptor γ (PPARγ), a nuclear receptor involved in several biological processes and in the maintenance of cellular homeostasis [62].

The analysis of the mutation sites in all the protein structures examined indicates that ~30% of them are buried from the solvent. Generally, the consequence of a mutation in the protein core are more likely to be deleterious, leading to protein misfolding [63]. If the mutated residue is on the surface of the protein, a minimal rearrangement of the exposed region may occur, however the global folding of the protein variant is maintained as well as its expression in the cell [64,65]. In either cases, missense mutations can lead to loss of function when generating unstable mutant proteins more susceptible to proteolysis, directly affecting binding affinity [66,67], or protein expression levels [68]. However, destabilizing mutations may also confer new functions when promoting interactions with new partners [56] or aggregation [69].

### 3.1. Effect on Protein Stability

Stability is a fundamental property of a protein [70,71] and it is one of the protein properties mostly affected by missense mutations [72,73]. About 80% of missense mutations associated with disease result in alteration of protein stability affecting it by several kcal/mol [72]. Therefore, it becomes essential to annotate whether a single nucleotide substitution associated to a given disease generates a SAV with a different stability [74].

Missense mutations may increase the conformation energy of the native state, destabilizing it and making the protein more prone to aggregation [69,75], which is a decisive event in some diseases characterized by aggregates of unfolded proteins [76]. However, in some cases, a mutation decreases the free energy of the native state, which might also turn out to be deleterious, if the increased stability limits the conformational changes important for the functionality of the protein. Generally, most disease-causing mutations are destabilizing [77,78,79]: if a mutation affects a stabilizing interaction within a folded protein, e.g., hydrophobic interactions or a network of hydrogen bonds, the native state may be destabilized. The loss of stability may be accompanied by loss of function [25], since most proteins need to be folded for functioning. The degree of destabilization is elevated for mutations introducing drastic changes, such as charged to neutral, or aromatic to aliphatic residue, that are often related to diseases. A consequence of the decrease in SAVs structural stability may be an increased proteolysis, which may lead to an insufficient amounts of that protein in the cells [80].

**Figure 2:**
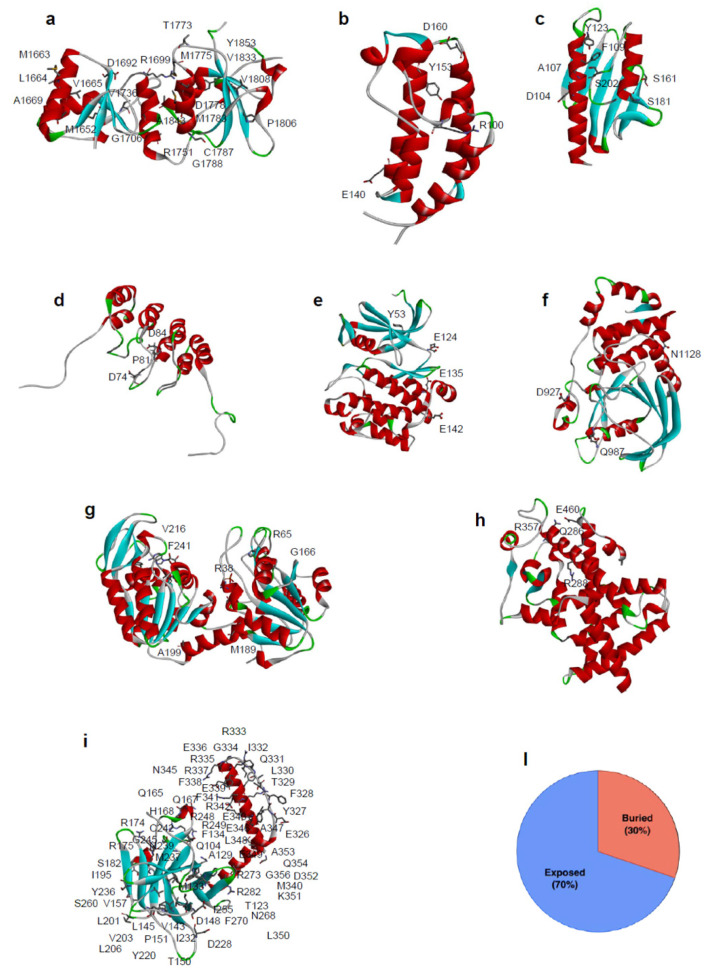
Location of SAVs analyzed in this review and their distribution based on the structural position of the mutated residues. Three-dimensional structure of the proteins analysed in this review are reported. (**a**) BRCA1 DNA repair associated protein (BRCA1) PDB code: 1JNX, (**b**) Bromodomain 2(1) (BRD) PDB code: 1X0J, (**c**) Frataxin (hFXN) PDB code: 1EKG, (**d**) p16 PDB code: 1DC2, (**e**) PIM-1 kinase PDB code: 1XWS, (**f**) Protein tyrosine phosphatase ρ (PTPρ) PDB code: 2OOQ, (**g**) Phosphoglycerate kinase 1 (PGK1) PDB code: 2XE7, (**h**) Peroxisome Proliferator Receptor γ (PPARγ) PDB code: 1PRG, (**i**) Tumor-protein p53 (p53) PDB code: 3Q01. Mutated residues in missense variants are depicted in stick. (**l**) Distribution of SAVs according to the structural position of the mutated residues for missense variants (30% of the mutation involved buried residues, 70% involved solvent exposed residues).

Folding studies and stability analysis have been performed on several missense variants of different proteins, measuring, by thermal or chemical unfolding, the impact of single amino acid substitution on the difference in Gibbs free energy value between the mutated and wild-type protein (ΔΔG_f_). The tumor suppressor p53 is one of the most frequently mutated proteins found in cancer [52,53,81]. Among the selected mutations of p53, many of them distributed throughout the core domain have been found to destabilize the protein between 1.2 and 4.8 kcal/mol (Figure 2i) [82]. Additionally, for BRCA1 (Figure 2a), several missense mutations were found to be highly destabilizing [83,84], particularly those buried in the hydrophobic core.

A further example of the impact of missense mutations on protein stability is represented by the case of hFXN, where 4 out of 8 SAVs show a significant alteration in the thermodynamic parameters (Figure 2c, Supplementary File S1).

In particular, the thermodynamic stability of the hFXN missense variants p.F109L, p.Y123S, p.S161I, and p.S181F is decreased in comparison to that of the wild type, whereas it is unchanged for p.D104G, where a charged polar residue is mutated into a small, uncharged amino acid, for p.S202F, where a polar residue is substituted by a hydrophobic one, and for p.A107V, where the two involved hydrophobic residues differ by their steric hindrance [85].

### 3.2. Effect on Protein Conformation

**Figure 3:**
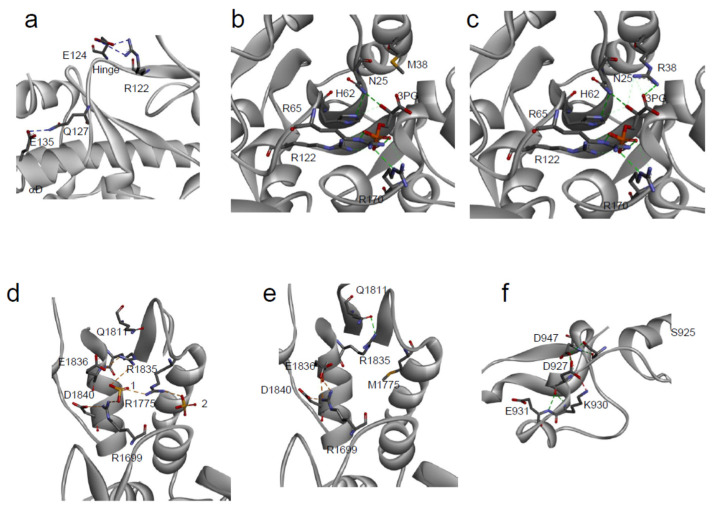
Local environment of SAVs. (**a**) PIM-1: Detailed view of the local structural environment around the mutated residue E135 [86]; (**b**,**c**) PGK1: detail of the 3 phosphoglycerate (3-PG) binding site in the variant p.R38M (**b**) in comparison with the wild-type (**c**) [87]; (**d**,**e**) BRCA1: Structural rearrangements of p.M1775R variant (**d**) in comparison with the wild-type, 1 and 2 are two solvent ions (**e**) [88]; (**f**) PTPρ: local environment of the mutated residue D927 [77].

Most of the SAVs in our dataset, for which protein conformational changes have been evaluated, e.g., by near-UV Circular Dichroism (CD), or by intrinsic fluorescence changes, show only minor perturbations in the tertiary structure arrangements. Nevertheless, in some SAVs, significant differences in protein conformation were observed, as in the case of p.E135K variant of PIM-1, whose near-UV CD and fluorescence emission spectra dramatically differ from that of the wild-type [86]. The residue E135 is in helix αD and forms a hydrogen bond with Q127, which is likely to be important in stabilizing this helix (Figure 2e and Figure 3a). Notably, a significant reduction in the protein activity was also observed for p.E135K: the mutated protein showed only about 3% of the protein kinase activity of the corresponding wild-type, as well as a decrease in activation energy, which suggests an increase in flexibility with respect to the wild-type counterpart [86].

The minor conformational changes, observed in SAVs by near-UV CD and fluorescence emission, can also be observed as minimal changes in their 3D structure. The crystal structure of PGK1 p.R38M somatic variant [87] is closely similar to that of the corresponding wild-type and only local differences can be detected. The residue R38 is placed in the N-terminal 3-phosphoglycerate binding domain, where it is important for substrate binding, and its correct positioning is required to react with ADP (Figure 3c).

Moreover, together with residues K215 and K219, R38 is critical for charge balancing of the transition state, directly interacting with the transferring phosphate group in the closed conformation of PGK1. Compared to the wild-type structure, the overall structure of p.R38M is conserved (Figure 2g and Figure 3b,c), with only minor differences between the two: at the level of α-helix 374–382, visible in the p.R38M variant but not in the wild-type, and in the position of the β-phosphate group, which in p.R38M points towards the helix (Figure 3b,c).

Despite the minimal changes in the p.R38M crystal structure, a dramatic effect of mutation on the kinetic parameters of PGK1 was observed; *K*_M_ increased from 0.40 to 3.15 mM and the turnover number strongly decreased from 89.8 to 7.2 × 10^−6^ s^−1^. This confirms that local and minimal changes in the protein structure, induced by a missense mutation, can lead to major alterations in protein function.

The impact of cancer-related missense mutations on protein structure can be dramatic, as demonstrated by the interesting example of the BRCA1 p.M1775R (Figure 3d). BRCA1 is involved in the regulation of multiple nuclear functions, including transcription, recombination, DNA repair, and checkpoint control, and is frequently mutated in cancer [89]. The p.M1775R variant of the C-terminal domain of BRCA1 (BRCT) cannot interact with histone deacetylases [90], the DNA helicase BACH1 [91], or with the transcriptional co-repressor CtIP [92,93]. The residue p.M1775 is largely buried and its substitution with an arginine residue creates a clustering of three positively charged residues (R1699, R1775 and R1835) (Figure 3d,e). In the wild-type native structure, R1699 participates in the sole conserved salt bridge of the inter-BRCT repeat, formed with a pair of carboxyl-terminal BRCT acidic residues, D1840 and E1836 (Figure 3e). R1835 normally participates in a hydrogen bonding network with Q1811, thereby helping to orient the β^1^-α^1^ loop (Figure 3c). In the p.M1775R variant, R1699 retains the salt bridge with D1840 but no longer contacts E1836 and instead coordinates an anion, R1835 rotates away from Q1811 and forms a new salt bridge with E1836 (Figure 3d) [84,88].

### 3.3. Effect on Protein Interactions

Approximately 60% of disease-associated SAVs show significant perturbations in the protein binding sites, resulting in complete loss of interactions and/or function [94,95,96]. In particular, if the mutated residue is essential in contributing to the interactions with partners [97,98,99,100], the binding affinity, as well as the binding specificity, would be dramatically affected, due to geometrical constraints and/or energetic effects [101,102,103,104]. For example, the deleterious mutations E330K and G352R of SMAD4, clustered near the SMAD4–SMAD3 interaction interface, are associated with juvenile polyposis [105,106]. This observation is in agreement with previous evidence associating the juvenile polyposis with the disruption of the signaling pathway TGFβ/SMAD which includes the interaction of SMAD4–SMAD3 [107,108].

Several SAVs exhibit alterations in their binding properties. An interesting example is represented by the BRDs, small helical interaction modules that specifically recognize acetylation sites in proteins. BRD2(1) (Figure 2b) and BRD3(2) SAVs show significant differences in their binding to two inhibitors of pharmacological interest, PFI-1 and JQ1, that may be related to the location of the missense mutations in proximity of a region important for the binding to acetylated peptides [109].

### 3.4. Effect on Protein Catalytic Activity

In the human population, 25% of the known SAVs show a significant modification of their biological function [76]. This percentage is mostly covered by mutations that occur in the active sites of enzymes or in the binding pockets of receptors [110,111]. Biochemical reactions are very sensitive to the precise geometry of the active sites [112,113,114]. Enzyme catalysis, however, does not depend just on a restricted number of crucial residues in the catalytic pocket, but also on several surrounding residues, important for ensuring the proper positioning of the substrates and cofactors into the active site. Therefore, mutations that occur on the residues located in the neighborhood of the active site, although not directly involved in the catalytic event, may also influence the enzyme activity [112,113], as in the case of PGK1 SAVs, discussed in Section 3.2 (Figure 3b). An interesting example of changes in enzyme tyrosine phosphatase activity, due to the presence of missense mutations, is represented by the case of PTPρ (Figure 2f), that belongs to the classical receptor type IIB family of protein tyrosine phosphatase and may act as a tumor suppressor [115]. Among the PTPρ SAVs identified in human cancer tissues, the missense variant p.D927G is almost completely inactive at 37°C [77]. This mutation involves a solvent exposed residue, distant from the catalytic site, and placed in a 4-residues turn between two coils, that connects different secondary structure regions through hydrogen bonds with three residues (D947, K930, and E931) (Figure 3f). The highly destabilizing D927G mutation may presumably alter the main chain flexibility, leading to local disorder, and thus affecting the stabilizing hydrogen bonds of residues in its proximity [77]. This SAV is an interesting example of the cumulative effect of a missense mutation on thermodynamic stability and function.

## 4. Computational Analysis of Protein Variants in Cancer-Related Genes

The large amount of protein variants collected in public databases and the limitations in the experimental methods stimulated the development of several tools for predicting their impact on protein stability and their pathogenic effect. Accordingly, early developed tools focus on the prediction of protein stability change by estimating the variation of free energy change (ΔΔG_f_) resulting from an amino acid substitution [116,117]. The majority of methods, which have been trained on ProTherm database [118] or on manually collected datasets [26], predict either the value or the sign (positive/negative) of the ΔΔG_f_. More recently, once large databases collecting pathogenic variants were made available, many binary classifiers have been implemented for predicting the impact of genetic variants on human health [119,120,121]. All available methods for predicting the impact of variants on protein stability or on protein pathogenicity rely on the various features extracted from protein sequence, structure, and evolutionary information. State-of-the-art methods of both types are currently used for protein engineering and for variant interpretation. In this section, we analyze the effect of the protein variants using computational approaches for predicting protein stability changes and pathogenicity, with the aim of estimating the role of protein stability on cancer mechanisms and the reliability of computational tools on this specific task.

### 4.1. Collection of the Protein Variant Datasets

**Figure 4:**
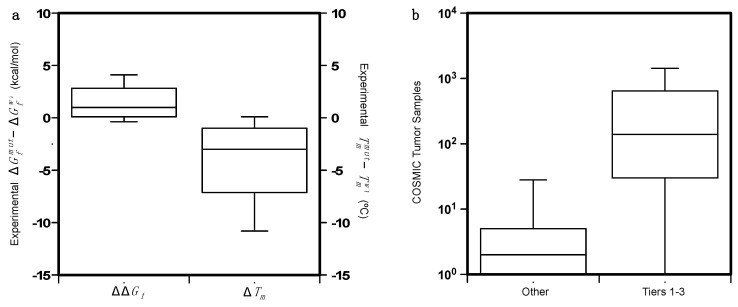
(**a**) Distributions of the of ΔΔG_f_ and Δ*T_m_* on the dataset of 164 protein variants. Δ*T_m_* is available only for 73 of them. (**b**) Comparison of the distributions of the COSMIC tumor samples for the putative cancer-driving variants (Tiers 1–3) and the benign variants (*Others*) annotated by the Cancer Mutation Census project.

In this work, we analyze a set of 164 missense variants from 11 proteins to understand the contribution of protein stability on the insurgence and progression of cancer (Table S1). Among the 11 proteins, 5 of them are mainly involved in regulation activities (BRD2, BRD3, BRD4, p16, and PPARγ), 4 have catalytic activities (PIM1, PGK1, FXN, and PTPρ) while the remaining 2 (p53 and BRCA1) are involved in many biological processes. The set collects all protein variants, for which the folding ΔΔG value was experimentally determined and whose genes are reported in the COSMIC database, either as Tier 1 genes, or with putative cancer-driving evidence. The folding ΔΔG_f_ is calculated as the difference between the folding ΔG of the mutant and wild-type proteins (ΔΔG_f_ = ΔG_f_*^mut^* − ΔG_f_*^wt^*), i.e., it is positive for destabilizing variants. When available, the variation of the melting temperature (Δ*T_m_* = *T_m_^mut^* − *T_m_^wt^*) was also collected. The distributions of the ΔΔG_f_ and Δ*T_m_* values are plotted in Figure 4a. The protein mutants are mapped on unique protein structures except in the case of p53, for which the DNA binding and oligomerization domains are considered separately. A subset of 97 variants from 9 proteins is obtained by matching our dataset with the data collected by the Cancer Mutation Census (CMC) project. This subset is composed of 24 putative cancer-driving variants annotated as “Tier 1–3” and 63 putative benign variants annotated as “*Other*”.

In Figure 4b we compared the distribution of the COSMIC tumor samples in which the putative cancer-driving variants (PCVs) and putative benign variants were detected. The somatic variants annotated by the CMC project are found in different tumor tissues. In particular, the hotspot mutants in the p53 DNA-binding region are detected in tumors from more than 30 tissues. The final list of the variants with their relative annotations and features are reported in Supplementary File S1.

### 4.2. Analysis of the Protein Variants

In this review we verified the possibility of using the available methods for predicting the impact of missense variants to identify key functional residues of the protein. We first evaluated the performance of a state-of-the-art method (FoldX [27]) in the prediction of ΔΔG_f_ resulting from an amino acid substitution. In the second step of the analysis, we predicted the pathogenicity of the selected variants to identify putative cancer-driving variants. For this task we used Meta-SNP [28], a meta prediction algorithm combining the output of 4 methods, namely PhD-SNP [122], PANTHER [123], SIFT [124], and SNAP [125]. The Meta-SNP output is considered as a proxy for predicting PCVs as reported in the Cancer Mutation Census (https://cancer.sanger.ac.uk/cmc (accessed on 1 May 2021)). For optimizing the prediction process on our set of cancer-associated genes we performed a 5-fold cross-validation procedure to select the best classification threshold. For a better characterization of the results, we also evaluated the importance of protein structure and evolutionary information in the detection of putative cancer-driving variants.

### 4.3. Predicting the Folding Free Energy Change of the Protein Variants

For each mutant in our dataset, we predicted the variation of the folding free energy (ΔΔG_f_) using FoldX, which is one of the most accurate methods for such a task [79]. For each mutant, we averaged the FoldX predictions on 10 models of the mutated structure. The predictions on the whole set of mutants are reported in Supplementary File S1. We then compared the predicted and the experimental ΔΔG_f_ values and calculated the performance on the set of variants, either grouped by proteins or on the whole set. In particular, we calculated three types of correlation (Pearson, Spermann, Kendall-Tau), and two error estimates, the Root Mean Square Error (RMSE) and the Mean Absolute Error (MAE). The results in Table S2 show that, on the whole set of 164 variants, FoldX achieves a Pearson correlation coefficient (*r_P_*) of 0.50 and a RMSE of 2.1 kcal/mol. This result can be improved by removing the variant p.G1788V of BRCA1 from the dataset. For that variant, FoldX predicts a ΔΔG_f_ of 15.2 kcal/mol, which is much higher than all other predicted values. Such a large predicted ΔΔG value is likely due to a limitation of FoldX, which in this case fails to identify a stable conformation of the protein mutant. After removing p.G1788V from the dataset, the *r_P_* increased to 0.56 and the RMSE decreased to 1.8 kcal/mol. On average we observed that the predicted ΔΔG values returned by FoldX tend to be larger than the experimental values. This behavior is probably due to the implementation of the FoldX algorithm which predicts the structure of the mutant protein only considering different rotamers of the amino acid side chains. Such a process might limit the ability of the tool to identify more stable conformations that could be obtained through the rearrangement of the backbone.

**Figure 5:**
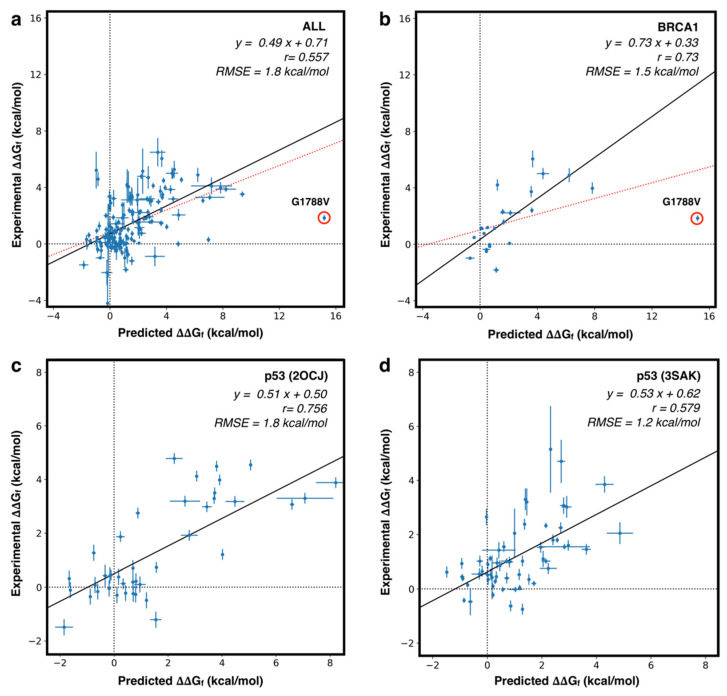
Scatter plot for predicted vs experimental ΔΔG_f_ values. (**a**) fitting on the whole set of 164 variants (red) and after removing the outlier BRCA1 p.G1788V (black). (**b**) Similar analysis performed on the whole set of variants in BRCA1 (red) and after removing BRCA1 p.G1788V variant (black). (**c**,**d**) fitting on the subset of variants in p53 core (2OCJ) and oligomeric (3SAK) domains, respectively. *r*: Pearson correlation coefficient. RMSE: Root Mean Square Error.

Further analysis was performed after grouping the variants by proteins and calculating the performance on the resulting protein subsets. By doing so, we observed for some proteins (both domains of p53, hFXN, PGK1, and PTPρ) a *r_P_* > 0.58. For other proteins (BRDs, PIM-1, and p16) with a smaller number of mutants (≤10), we observed lower or negative correlation coefficients. The scatter plots, showing the correlation between predicted and experimental ΔΔG_f_ values for the whole set of proteins, or for the proteins with the highest number of mutants (p53 and BRCA1), are shown in Figure 5. Another interesting analysis consists in the prediction of highly destabilizing variants (ΔΔG_f_ > 2 kcal/mol). In this case, we have used FoldX as a binary classifier, optimizing a threshold on its output.

The optimization procedure, based on balancing the true positive and true negative rates, shows that FoldX can achieve an overall accuracy of 77% and a Matthews Correlation Coefficient (MCC) of 0.55 when a prediction threshold of ~1.2 kcal/mol is considered for the whole set of 164 variants. This method shows a good performance also when considering protein-specific thresholds. Indeed, for the subset of proteins with the highest number of mutants (p53 and BRCA1), the performance in the classification task reached MCC = 0.78 and AUC (Area Under the ROC curve) = 0.95 for the DNA binding domain of p53, or MCC = 0.67 and AUC = 0.90 for BRCA1. All performance measures in the classification task are summarized in Table S3.

In general, our analysis confirms that, on average, the predicted and experimental ΔΔG_f_ correlate well, and that the FoldX prediction can be used to estimate the impact of mutations of protein stability, in spite of the fact that the prediction error still remains ~2.0 kcal/mol. To partially address this limitation, the methods for ΔΔG_f_ prediction can be used as binary classifiers to detect highly destabilizing protein variants.

### 4.4. Predicting the Pathogenicity of Protein Variants

In the last decade, several methods have been developed for predicting the pathogenicity of variants. In general, those approaches are binary classifiers, based on the analysis of evolutionary conservation. The idea behind these tools is based on the observation that mutations occurring in highly conserved regions of the protein are more likely to be pathogenic than mutations in variable regions. In the case of cancer-associated variants, the validation of the predictive methods is a difficult task due to the lack of curated sets of annotated variants. To address this issue, the COSMIC curators are annotating the somatic mutations in the Cancer Mutation Census (CMC) dataset [19]. Currently, the CMC contains ~3 million missense variants, only ~0.1% of which were curated. Using such annotation, we analyzed the prediction of Meta-SNP, an algorithm combining the output of 4 methods, on our dataset. Initially, we analyzed the relationship between the experimental ΔΔG_f_ and the variant pathogenicity score returned by Meta-SNP, to test its performance in the detection of highly destabilizing variants (ΔΔG_f_ > 2 kcal/mol). Setting the optimized classification threshold to 0.66, we found that Meta-SNP reaches an accuracy of 73% and a Pearson correlation coefficient of 0.40 in the classification of highly destabilizing variants. We also estimated the performance of Meta-SNP in the prediction of putative cancer-driving variants (PCVs), assuming that missense variants, annotated as “Other” in the CMC database, can be classified as benign and variants in CMC annotated as classes 1–3 (Tier 1–3) can be considered putative cancer-driving variants (PCVs). For a more stringent test we calculated the performance of Meta-SNP by removing from the dataset 15 mutations used for the training of the method. Our results show that, for the subset of 82 variants annotated in CMC, by using a classification threshold of 0.71, Meta-SNP is able to predict PCVs with 77% accuracy and a Matthews correlation coefficient of 0.37.

The high fraction of false positives in the prediction of highly destabilizing variants may indicate the presence of pathogenicity mechanisms alternative to the loss of stability, while the high rate of false positives in the prediction of PCVs can be due to incorrect and/or incomplete protein variants annotation.

Although the Meta-SNP predictions result in a high fraction of false positive, the PCVs, annotated with 1 to 3 in the CMC database, are enriched in the variants with folding ΔΔG_f_ > 2 kcal/mol with respect to the subset of CMC variant annotated as “Other”. Indeed, the relative p-value calculated by the Fisher test is <0.03.

Furthermore, the comparison of the distributions of the Meta-SNP output for Tier 1–3 and “Other” variants reveals a significant difference. The average values of the distributions of Meta-SNP outputs for Tier 1–3 and “Other” variants are 0.71 and 0.47, respectively. This difference is statistically significant, corresponding to a Kolmogorov-Smirnov *p*-value < 10^−4^.

Finally, we also tested the performance of FoldX in predicting PCVs. Our analysis revealed that, selecting a predicted ΔΔG_f_ threshold of 2.7 kcal/mol, FoldX is able to identify Tier 1–3 variants with 73% overall accuracy and a Matthews correlation coefficient of 0.33. The comparison of the results shows that a predictor of putative pathogenic variants (Meta-SNP) is performing better than a method designed to predict folding ΔΔG_f_ (FoldX) in the detection of PCVs.

The performances of Meta-SNP and FoldX in the prediction of destabilizing and putative cancer-driving variants are summarized in Table S4.

### 4.5. Analysis of the Prediction on the Basis of the Amino Acid Accessibility and Conservation

In the previous sections, we have shown that protein stability of cancer-related genes can be predicted with a good level of confidence using dedicated computational tools like FoldX. We have also observed that the pathogenicity score, calculated through a consensus method, correlates with protein stability data and with phenotypic data. Nevertheless, the prediction of PCVs, starting from protein stability predictions, is a more complex task. To this end, more experimental data on the stability of cancer proteins and their variants, and a higher level of curation of the existing databases on cancer protein variants would be needed. As an in-silico alternative for estimating the impact of protein variants on the stability and phenotypic levels, we used Meta-SNP, which is one of the state-of-the-art methods that best predict the protein variant pathogenic potential. To better analyze the results obtained by Meta-SNP, we calculated the distributions of solvent accessibility and conservation scores of the wild-type residues for the subset of highly destabilizing and PCVs.

In detail, for each mutated site we calculated the relative solvent accessibility (RSA) of the mutated residues and the frequency of the wild-type residue in the multiple sequence alignment (*f_WT_*) of possible homologs of the mutated protein.

**Figure 6:**
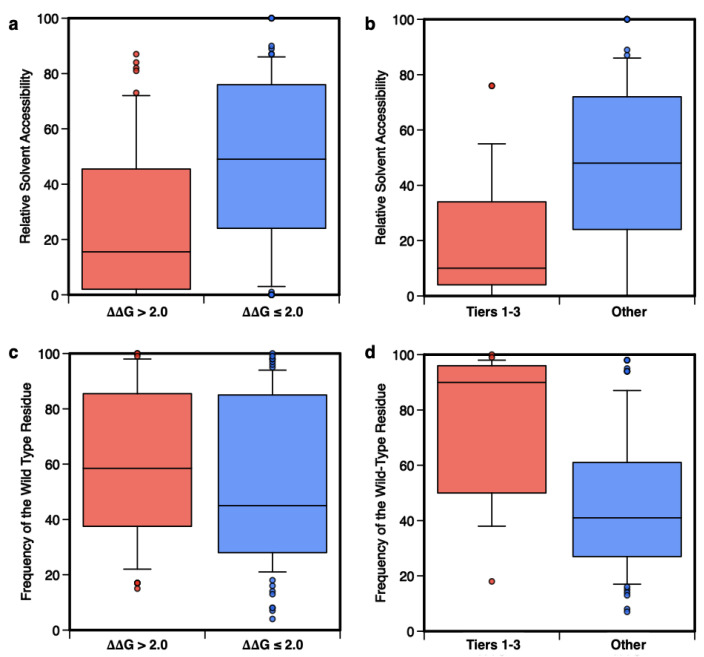
Distributions of the Relative Solvent Accessibility (RSA) and frequency of the wild-type residue (*f_wt_*) in the multiple sequences of homolog proteins. The distributions of RSA and *f_wt_* calculated for the subsets of 53 highly destabilizing variants (ΔΔG_f_ > 2.0 kcal/mol) compared to the remaining 111 variants (ΔΔG_f_ ≤ 2.0 kcal/mol) are shown in panels (**a**,**c**). In panels (**b**,**d**) the same distributions are plotted for the subsets of 24 putative cancer-driving (Tier 1–3) or 73 benign (‘Other’) variants.

As described in supplementary materials, the RSA was calculated by normalizing the solvent accessibility calculated with the DSSP program [126] and the *f_WT_* was returned as part of the output of the Meta-SNP server (http://snps.biofold.org/meta-snp, accessed on 1 May 2021). In the first part of our analysis, we compared the RSA values for the subset of highly destabilizing variants (ΔΔG > 2.0 kcal/mol) and the remaining ones, showing that for RSA ≤ 0.2 there is little overlap between the two distributions (Figure 6a). In the same range of RSA (Figure 6b), the PCVs (Tiers 1–3) can be easily discriminated from benign ones (“Other”). In both cases, using the Kolmogorov-Smirnov test to estimate the statistical difference between the two subsets in Figs. 6a and 6b, we obtained *p*-values < 10^−3^. In particular, Figure 6b shows that the majority of PCVs (~58%) are occurring in buried regions (RSA ≤ 0.2), while ~75% of putative benign variants are in exposed regions (RSA > 0.2). The fraction of PCVs in exposed regions, which we found in our dataset, is higher than the value reported for pathogenic variants [63], nevertheless, due to the reduced size of our dataset, such a difference is not statistically significant.

In the second part of this analysis, different results are observed when the distributions of *f_WT_* are compared. Figure 6c shows that conservation is not a strong feature for the classification of highly destabilizing variants, while it is essential for the prediction of PCVs. Indeed, for *f_WT_* > 50% the distributions of Tier 1–3 and “Other” variants have little overlap (Figure 6d). The comparison between the results in Figure 6c,d indicates that destabilization and conservation may indeed serve the pathogenicity prediction task as reciprocally integrating features [25].

**Figure 7:**
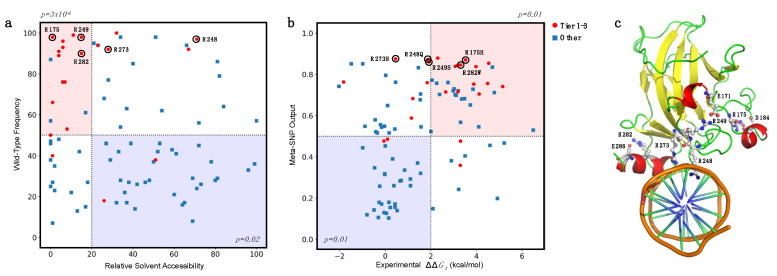
Enrichment and depletion of putative cancer-driving variants (Tier 1–3) in different subgroups. (**a**) Enrichment of Tier 1–3 variants in mutated sites with Relative Solvent Accessibility ≤20% and frequency of the wild-type residue > 50% (light red). (**b**) Enrichment of Tier 1–3 variants in the subset of mutants with experimental ΔΔG_f_ > 2.0 kcal/mol and Meta-SNP output >0.5 (light red). In both cases, the opposite regions (light blue) are depleted of Tier 1–3 variants. In (**a**,**b**), the hotspot mutants R175, R248, R249, R273, and R282 are highlighted with black circles. (**c**) Hotspot sites in the p53 structure in interaction with DNA (PDB:1TUP). Residues R248 and R273 interact with DNA. Residues R175, R249, and R282 are likely to stabilize the protein structure by forming salt bridge interactions with D162, E171, and E286, respectively.

A further interesting analysis can be performed by considering the distribution in two dimensions of the RSA and *f_WT_* together for Tiers 1–3 and “Other” variants. In Figure 7a we observed an enrichment for Tiers 1–3 variants in the buried (RSA < 20%) and conserved residues (*f_WT_* > 50%), with a corresponding p-value of 3 × 10^−6^, obtained by considering a binomial distribution with a success probability of 0.247. On the opposite side of the plot (RSA ≥ 20% and *f_WT_* ≤ 50%), we observed a depletion of PCVs (*p*-value = 0.02). Finally, we performed a similar analysis by combining the experimental ΔΔG_f_ with the Meta-SNP predictions (Figure 7b).

If we considered the subset of highly destabilizing (ΔΔG_f_ > 2.0 kcal/mol) and predicted pathogenic (Meta-SNP output > 0.5) variants, we found an enrichment in Tier 1–3 variants with corresponding p-value of 0.01. On the opposite side of the plot, we observed a depletion of Tier 1–3 variants, again with a *p*-value of 0.01.

These observations confirm the hypothesis that relative solvent accessibility and amino acid conservation are important features for predicting the impact of amino acid substitution in terms of protein stability and pathogenicity. Furthermore, the combination of the experimental ΔΔG_f_ and the predicted pathogenicity of variants allows to select a subset of variants with a significantly high probability of having a deleterious phenotypic effect.

In particular, focusing on a subset of five hotspot sites of p53 [127,128], we observed that R248 and R273 are directly interacting with the DNA, in agreement with their high RSA, while R175, R249, and R282 (low RSA) are surrounding the DNA binding site (Figure 7c). These structural aspects, combined with our predictions, support the hypothesis that the p.R248Q and p.R273H variants (with high pathogenicity score but low ΔΔG_f_) have a direct impact on the protein function of DNA-interaction, while p.R175H and p.R282W (with both high pathogenicity score and high ΔΔG_f_) destabilize the p53 structure. An intermediate case is p.R249S, which shows a variation of folding free energy of ~2 kcal/mol and a low RSA. Similar to R175 and R282, the presence of an oppositely charged residue (E171) in the proximity of R249 suggests that a mutation in this site can indeed reduce the stability of p53, due to a missing salt bridge interaction. Although a significant difference between predicted and experimental ΔΔG_f_ values (RMSE = 3.2 kcal/mol) is observed for the five hotspots, similar results are obtained when combining the Meta-SNP output with the predicted ΔΔG_f_ (Figure S1). Our analysis can be compared with the experimental data on DNA-binding affinity of the p53 mutants [82]. The data show that among the five hotspots cancer mutants shown in Figure 7, the three of them with low impact on p53 stability (p.R248Q, p.R249S, and p.R273H with ΔΔG_f_ ≤ 2.0 kcal/mol) had no detectable binding affinity with the gadd45 promoter DNA (0% with respect to the wild type). This observation supports the hypothesis that protein–DNA interactions may play an important role in the cancer-inducing mechanism of the mutated p53. By analogy, a similar case of compromised protein–DNA or protein–protein interactions might turn out to hold for other cancer-associated mutants, which might not be in the ‘highly destabilizing’ category. The above observation is also in agreement with the possible roles hotspots cancer p53 mutants are considered to play as gain-of-function effectors [127,128], not only for the ‘contact’ mutants p.R248Q and p.R273H, but also for the ‘conformational’ mutants p.R175H, p.R249S, and p.R282W, since an altered p53 binding energy landscape can shift the mutated cells to different functionalities. Furthermore, the data shown in Figure 7 are consistent with the fact that also destabilizing variants can have gain-of-function characteristics, possibly through altered protein–protein interactions [56].

## 5. Conclusions and Future Perspectives

Single nucleotide variations in DNA, resulting in amino acid substitutions (SAVs), can lead to changes in the protein stability [129,130] or to alterations at the structural level, which may have an influence on protein function. In the present work, we have focused our analysis on those SAVs occurring in cancer-related genes and reported in COSMIC, for which their impact on protein stability had been experimentally determined. Cancer is a complex multigenic disease, in which more than one SAV, occurring on different proteins, may act in coordination. For a deeper understanding of the mechanisms of cancer at a molecular level, leading to the identification of personalized therapeutic strategies, a detailed analysis of the properties of the variants reported in the publicly available databases is essential. Experimental studies on the effects of missense mutations on protein conformation, stability, function, and interactions have been carried out in detail just on a limited number of nsSNVs, e.g., p53 and BRCA1, and few three-dimensional structures of protein variants are available in comparison with the large amount of SAVs reported in COSMIC. It is only through an intensified dialogue between the experimental and the computational efforts that one can expect to contribute ever more relevant information for diagnosis. These studies can be helpful in following drug discovery design projects and pharmacological studies for searching the most useful treatment, to ensure the most precise patient care [131,132].

In this context, the computational methods, developed for predicting the effect of variants on protein stability and their pathogenicity, represent valuable tools to narrow down the number of expensive and time-consuming experimental procedures to be employed [10]. The role of experimental biochemical assays, in combination with computational analysis, is important for the selection of representative case studies, aimed at providing a more complete picture of the molecular mechanisms of cancer, and for a better customization of the available tools. In this sense, the Critical Assessment of Genome Interpretation (CAGI) represents a good example of how a common task framework can help to reach significant gains in the prediction of the phenotypic impacts of genomic variations [133]. In 2019, we proposed a new computing experiment, focusing on the prediction of the variation of the free energy change induced by hFXN missense variants [85]. The assessment of the predictions submitted for the hFXN, and other challenges focusing on clinical applications, confirmed that the state-of-the-art methods for predicting the variation of protein stability change upon mutation are achieving a good level of performance, while methods for predicting pathogenic variants reached a good level of performance on challenges focusing on possible clinical applications [134,135,136]. The global effects of missense mutations on proteins can be highlighted from the analysis of the thermodynamic parameters of stability, distinguishing neutral mutations from destabilizing or stabilizing ones. Accordingly, we assessed the performances of two state-of-the-art methods (FoldX and Meta-SNP) in the prediction of the impact of missense mutation on protein stability and their implication in cancer. After an optimization process, based on the selection of appropriate classification thresholds, the computing methods achieved good performances in both tasks. Our analysis shows that the combination of methods for predicting ΔΔG_f_ or pathogenicity is a good strategy for estimating the impact of variants at structural and functional levels. Indeed, the enrichment of Tier 1–3 variants in the subset of highly destabilizing and pathogenic variants is consistent with the most common pathogenic mechanism being the destabilization of the structure, which results in loss of function. Nevertheless, the possibility of alternative pathogenic mechanisms based on gain of function, and the presence of a not negligible number of false positives, requires a more in-depth analysis of the main structural and evolutionary features which represent the basis of our predictions. Based on the observation that a large fraction of Tier 1–3 variants corresponds to conserved sites in the core of the protein, it would be as much informative to focus both on the role of those highly conserved residues which do occur on the exposed regions of the protein, and on the putative destabilizing variants found in non-conserved regions. The first group of variants may affect functional sites important for protein–protein and protein–DNA interactions. The second class of mutants may have a significant impact on the local-to-global structure of the protein, generating, or shifting the equilibrium towards, new functional conformations. For example, a plethora of evidence confirms that mutant p53 proteins not only lose their tumor-suppressive function and acquire dominant-negative activities, but also gain new oncogenic properties [137] by affecting the transcription of various genes, as well as by protein–protein interactions with transcription factors and other effectors [56]. A recent review emphasizes the cellular existence of p53 as a highly complex and dynamic conformational ensemble, and reports experimental evidence on mutations shown to induce proteoforms of p53 [138], among which the highly destabilizing hotspot mutations of Arg337 in the oligomerization domain [139,140]. Nevertheless, the limited experimental data on alternative functional conformations does not allow to draw a general conclusion [141].

In the complex scenario of cancer onset, progression, and invasion, a single amino acid substitution may be plausibly considered as an adaptive attempt by nature, and chance, to produce a more functional protein in the neoplastic disorder. Cancer may be considered an adaptive evolutionary process [32,142], in which the accumulation of random genetic changes in cells and tissues is mainly driven by the necessity of cells to proliferate maximizing fitness based on environmental circumstances [29]. In this regard, it should be noted that few missense mutations in cancer-related proteins result in stabilizing effects on the native protein or are neutral, not affecting protein stability. Many of the SAVs studied, indeed, show changes of their tertiary arrangements with local alterations that do not induce large conformational changes in the protein 3D structure. These local changes can lead to differences in protein flexibility that may possibly influence the protein interactions network, particularly when the point mutation involves a solvent exposed residue. The tertiary structure changes of SAVs may alter the binding to natural partners and perturb the interactions with ligands and/or inhibitors. Structural analysis in solution combined with 3D structure analysis of missense variants may help to get a deeper insight into the molecular mechanism underlying complex diseases. This information regarding cancer-related genes may also provide useful clues for the identification of diagnostic markers and for optimization of personalized treatments, particularly for those variants not accompanied by significant changes in stability. For a genomic and molecular profiling of the individual, the research in biomedicine might increasingly focus on the integration of experimental and computational techniques, leading to the development of more effective treatment strategies.

## Figures and Tables

**Table 1:** Selected list of the databases of single nucleotide variants.

Variant Database	Database Description	Web Site	Reference
**ClinVar**	ClinVar aggregates information about genomic variation and its relationship to human health.	http://www.ncbi.nlm.nih.gov/clinvar (accessed on 1 May 2021)	[1]
**COSMIC**	Manually curated resource of somatic mutation of human cancers.	https://cancer.sanger.ac.uk/cosmic (accessed on 1 May 2021)	[19]
**dbSNP**	Database of human single nucleotide variations, microsatellites, and small insertions and deletions for both common variations and clinical mutations.	http://www.ncbi.nlm.nih.gov/snp (accessed on 1 May 2021)	[20]
**UniProt** **humsvar**	Lists of missense variants annotated in UniProtKB/Swiss-Prot human entries. The variant classification should not be considered for clinical and diagnostic use.	https://www.uniprot.org/docs/humsavar (accessed on 1 May 2021)	[21]
**ICGC Data Portal**	The ICGC Data Portal provides many tools for visualizing, querying, and downloading cancer data.	https://dcc.icgc.org/ (accessed on 1 May 2021)	[22]
**OMIM**	Online Catalog of Human Genes and Genetic Disorders.	http://www.omim.org (accessed on 1 May 2021)	[23]
**GDC**	The GDC Data Portal provides access to GDC harmonized data as well as an archive of legacy data from TCGA and other NCI programs.	https://portal.gdc.cancer.gov/ (accessed on 1 May 2021)	[24]

## Data Availability

The data presented in this study are available in Supplementary File S1.

## Supplementary Materials

The following are available online at https://www.mdpi.com/article/10.3390/ijms22115416/s1.

## References

[1] Landrum M.J., Lee J.M., Benson M., Brown G., Chao C., Chitipiralla S., Gu B., Hart J., Hoffman D., Hoover J. (2016). ClinVar: Public Archive of Interpretations of Clinically Relevant Variants. Nucleic Acids Res..

[2] Karczewski K.J., Francioli L.C., Tiao G., Cummings B.B., Alföldi J., Wang Q., Collins R.L., Laricchia K.M., Ganna A., Birnbaum D.P. (2020). The Mutational Constraint Spectrum Quantified from Variation in 141,456 Humans. Nature.

[3] Abecasis G.R., Auton A., Brooks L.D., DePristo M.A., Durbin R.M., Handsaker R.E., Kang H.M., Marth G.T., McVean G.A., 1000 Genomes Project Consortium (2012). An Integrated Map of Genetic Variation from 1092 Human Genomes. Nature.

[4] Collins F.S., Guyer M.S., Charkravarti A. (1997). Variations on a Theme: Cataloging Human DNA Sequence Variation. Science.

[5] HapMap Consortium (2003). The International HapMap Project. Nature.

[6] Kruglyak L., Nickerson D.A. (2001). Variation Is the Spice of Life. Nat. Genet..

[7] Cheng N., Li M., Zhao L., Zhang B., Yang Y., Zheng C.H., Xia J. (2019). Comparison and Integration of Computational Methods for Deleterious Synonymous Mutation Prediction. Brief. Bioinform..

[8] Zhou T., Zhang Y., Macchiarulo A., Yang Z., Cellanetti M., Coto E., Xu P., Pellicciari R., Wang L. (2010). Novel Polymorphisms of Nuclear Receptor SHP Associated with Functional and Structural Changes. J. Biol. Chem..

[9] Ancien F., Pucci F., Godfroid M., Rooman M. (2018). Prediction and Interpretation of Deleterious Coding Variants in Terms of Protein Structural Stability. Sci. Rep..

[10] Malhotra S., Alsulami A.F., Heiyun Y., Ochoa B.M., Jubb H., Forbes S., Blundell T.L. (2019). Understanding the Impacts of Missense Mutations on Structures and Functions of Human Cancer-Related Genes: A Preliminary Computational Analysis of the COSMIC Cancer Gene Census. PLoS ONE.

[11] MacArthur D.G., Manolio T.A., Dimmock D.P., Rehm H.L., Shendure J., Abecasis G.R., Adams D.R., Altman R.B., Antonarakis S.E., Ashley E.A. (2014). Guidelines for Investigating Causality of Sequence Variants in Human Disease. Nature.

[12] Lappalainen T., Scott A.J., Brandt M., Hall I.M. (2019). Genomic Analysis in the Age of Human Genome Sequencing. Cell.

[13] Buniello A., MacArthur J.A.L., Cerezo M., Harris L.W., Hayhurst J., Malangone C., McMahon A., Morales J., Mountjoy E., Sollis E. (2019). The NHGRI-EBI GWAS Catalog of Published Genome-Wide Association Studies, Targeted Arrays and Summary Statistics 2019. Nucleic Acids Res..

[14] Flannick J., Florez J.C. (2016). Type 2 Diabetes: Genetic Data Sharing to Advance Complex Disease Research. Nat. Rev. Genet..

[15] Liu Y., Easton J., Shao Y., Maciaszek J., Wang Z., Wilkinson M.R., McCastlain K., Edmonson M., Pounds S.B., Shi L. (2017). The Genomic Landscape of Pediatric and Young Adult T-Lineage Acute Lymphoblastic Leukemia. Nat. Genet..

[16] Bailey M.H., Tokheim C., Porta-Pardo E., Sengupta S., Bertrand D., Weerasinghe A., Colaprico A., Wendl M.C., Kim J., Reardon B. (2018). Comprehensive Characterization of Cancer Driver Genes and Mutations. Cell.

[17] (2020). ICGC/TCGA Pan-Cancer Analysis of Whole Genomes Consortium Pan-Cancer Analysis of Whole Genomes. Nature.

[18] Nussinov R., Tsai C.-J., Jang H. (2019). Why Are Some Driver Mutations Rare?. Trends Pharmacol. Sci..

[19] Tate J.G., Bamford S., Jubb H.C., Sondka Z., Beare D.M., Bindal N., Boutselakis H., Cole C.G., Creatore C., Dawson E. (2019). COSMIC: The Catalogue Of Somatic Mutations In Cancer. Nucleic Acids Res..

[20] Sherry S.T., Ward M.H., Kholodov M., Baker J., Phan L., Smigielski E.M., Sirotkin K. (2001). DbSNP: The NCBI Database of Genetic Variation. Nucleic Acids Res..

[21] Mottaz A., David F.P., Veuthey A.L., Yip Y.L. (2010). Easy Retrieval of Single Amino-Acid Polymorphisms and Phenotype Information Using SwissVar. Bioinformatics.

[22] Zhang J., Baran J., Cros A., Guberman J.M., Haider S., Hsu J., Liang Y., Rivkin E., Wang J., Whitty B. (2011). International Cancer Genome Consortium Data Portal--a One-Stop Shop for Cancer Genomics Data. Database Oxf..

[23] Amberger J.S., Bocchini C.A., Scott A.F., Hamosh A. (2019). OMIM.Org: Leveraging Knowledge across Phenotype-Gene Relationships. Nucleic Acids Res..

[24] Grossman R.L., Heath A.P., Ferretti V., Varmus H.E., Lowy D.R., Kibbe W.A., Staudt L.M. (2016). Toward a Shared Vision for Cancer Genomic Data. N. Engl. J. Med..

[25] Stein A., Fowler D.M., Hartmann-Petersen R., Lindorff-Larsen K. (2019). Biophysical and Mechanistic Models for Disease-Causing Protein Variants. Trends Biochem. Sci..

[26] Sanavia T., Birolo G., Montanucci L., Turina P., Capriotti E., Fariselli P. (2020). Limitations and Challenges in Protein Stability Prediction upon Genome Variations: Towards Future Applications in Precision Medicine. Comput. Struct. Biotechnol. J..

[27] Schymkowitz J., Borg J., Stricher F., Nys R., Rousseau F., Serrano L. (2005). The FoldX Web Server: An Online Force Field. Nucleic Acids Res..

[28] Capriotti E., Altman R.B., Bromberg Y. (2013). Collective Judgment Predicts Disease-Associated Single Nucleotide Variants. BMC Genom..

[29] Gatenby R.A., Brown J. (2017). Mutations, Evolution and the Central Role of a Self-Defined Fitness Function in the Initiation and Progression of Cancer. Biochim. Biophys. Acta Rev. Cancer.

[30] Sackton T.B., Lazzaro B.P., Schlenke T.A., Evans J.D., Hultmark D., Clark A.G. (2007). Dynamic Evolution of the Innate Immune System in Drosophila. Nat. Genet..

[31] Cairns J. (1975). Mutation Selection and the Natural History of Cancer. Nature.

[32] Greaves M., Maley C.C. (2012). Clonal Evolution in Cancer. Nature.

[33] Nowell P.C. (1976). The Clonal Evolution of Tumor Cell Populations. Science.

[34] Lee B., Tran B., Hsu A.L., Taylor G.R., Fox S.B., Fellowes A., Marquis R., Mooi J., Desai J., Doig K. (2018). Exploring the Feasibility and Utility of Exome-Scale Tumour Sequencing in a Clinical Setting. Intern. Med. J..

[35] Zhang Y., Manjunath M., Yan J., Baur B.A., Zhang S., Roy S., Song J.S. (2019). The Cancer-Associated Genetic Variant Rs3903072 Modulates Immune Cells in the Tumor Microenvironment. Front. Genet..

[36] Milanese J.-S., Wang E. (2019). Germline Mutations and Their Clinical Applications in Cancer. Breast Cancer Manag..

[37] Chan S.H., Lim W.K., Ishak N.D.B., Li S.-T., Goh W.L., Tan G.S., Lim K.H., Teo M., Young C.N.C., Malik S. (2017). Germline Mutations in Cancer Predisposition Genes Are Frequent in Sporadic Sarcomas. Sci. Rep..

[38] Hu C., Hart S.N., Polley E.C., Gnanaolivu R., Shimelis H., Lee K.Y., Lilyquist J., Na J., Moore R., Antwi S.O. (2018). Association Between Inherited Germline Mutations in Cancer Predisposition Genes and Risk of Pancreatic Cancer. JAMA.

[39] Meric-Bernstam F., Brusco L., Daniels M., Wathoo C., Bailey A.M., Strong L., Shaw K., Lu K., Qi Y., Zhao H. (2016). Incidental Germline Variants in 1000 Advanced Cancers on a Prospective Somatic Genomic Profiling Protocol. Ann. Oncol. Off. J. Eur. Soc. Med. Oncol..

[40] Schrader K.A., Cheng D.T., Joseph V., Prasad M., Walsh M., Zehir A., Ni A., Thomas T., Benayed R., Ashraf A. (2016). Germline Variants in Targeted Tumor Sequencing Using Matched Normal DNA. JAMA Oncol..

[41] Seifert B.A., O’Daniel J.M., Amin K., Marchuk D.S., Patel N.M., Parker J.S., Hoyle A.P., Mose L.E., Marron A., Hayward M.C. (2016). Germline Analysis from Tumor-Germline Sequencing Dyads to Identify Clinically Actionable Secondary Findings. Clin. Cancer Res. Off. J. Am. Assoc. Cancer Res..

[42] Mandelker D., Zhang L., Kemel Y., Stadler Z.K., Joseph V., Zehir A., Pradhan N., Arnold A., Walsh M.F., Li Y. (2017). Mutation Detection in Patients with Advanced Cancer by Universal Sequencing of Cancer-Related Genes in Tumor and Normal DNA vs Guideline-Based Germline Testing. JAMA.

[43] Bertelsen B., Tuxen I.V., Yde C.W., Gabrielaite M., Torp M.H., Kinalis S., Oestrup O., Rohrberg K., Spangaard I., Santoni-Rugiu E. (2019). High Frequency of Pathogenic Germline Variants within Homologous Recombination Repair in Patients with Advanced Cancer. NPJ Genom. Med..

[44] Qing T., Mohsen H., Marczyk M., Ye Y., O’Meara T., Zhao H., Townsend J.P., Gerstein M., Hatzis C., Kluger Y. (2020). Germline Variant Burden in Cancer Genes Correlates with Age at Diagnosis and Somatic Mutation Burden. Nat. Commun..

[45] Kampmeyer C., Nielsen S.V., Clausen L., Stein A., Gerdes A.-M., Lindorff-Larsen K., Hartmann-Petersen R. (2017). Blocking Protein Quality Control to Counter Hereditary Cancers. Genes. Chromosomes Cancer.

[46] Nielsen S.V., Poulsen E.G., Rebula C.A., Hartmann-Petersen R. (2014). Protein Quality Control in the Nucleus. Biomolecules.

[47] Kriegenburg F., Jakopec V., Poulsen E.G., Nielsen S.V., Roguev A., Krogan N., Gordon C., Fleig U., Hartmann-Petersen R. (2014). A Chaperone-Assisted Degradation Pathway Targets Kinetochore Proteins to Ensure Genome Stability. PLoS Genet..

[48] Casadio R., Vassura M., Tiwari S., Fariselli P., Luigi Martelli P. (2011). Correlating Disease-Related Mutations to Their Effect on Protein Stability: A Large-Scale Analysis of the Human Proteome. Hum. Mutat..

[49] Matreyek K.A., Starita L.M., Stephany J.J., Martin B., Chiasson M.A., Gray V.E., Kircher M., Khechaduri A., Dines J.N., Hause R.J. (2018). Multiplex Assessment of Protein Variant Abundance by Massively Parallel Sequencing. Nat. Genet..

[50] Nielsen S.V., Stein A., Dinitzen A.B., Papaleo E., Tatham M.H., Poulsen E.G., Kassem M.M., Rasmussen L.J., Lindorff-Larsen K., Hartmann-Petersen R. (2017). Predicting the Impact of Lynch Syndrome-Causing Missense Mutations from Structural Calculations. PLoS Genet..

[51] Ahner A., Nakatsukasa K., Zhang H., Frizzell R.A., Brodsky J.L. (2007). Small Heat-Shock Proteins Select DeltaF508-CFTR for Endoplasmic Reticulum-Associated Degradation. Mol. Biol. Cell.

[52] Bykov V.J.N., Eriksson S.E., Bianchi J., Wiman K.G. (2018). Targeting Mutant P53 for Efficient Cancer Therapy. Nat. Rev. Cancer.

[53] Chen S., Wu J.-L., Liang Y., Tang Y.-G., Song H.-X., Wu L.-L., Xing Y.-F., Yan N., Li Y.-T., Wang Z.-Y. (2021). Arsenic Trioxide Rescues Structural P53 Mutations through a Cryptic Allosteric Site. Cancer Cell.

[54] Li Y., Zhang Y., Li X., Yi S., Xu J. (2019). Gain-of-Function Mutations: An Emerging Advantage for Cancer Biology. Trends Biochem. Sci..

[55] Li X.-H., Babu M.M. (2018). Human Diseases from Gain-of-Function Mutations in Disordered Protein Regions. Cell.

[56] Stein Y., Rotter V., Aloni-Grinstein R. (2019). Gain-of-Function Mutant P53: All the Roads Lead to Tumorigenesis. Int. J. Mol. Sci..

[57] Miki Y., Swensen J., Shattuck-Eidens D., Futreal P.A., Harshman K., Tavtigian S., Liu Q., Cochran C., Bennett L.M., Ding W. (1994). A Strong Candidate for the Breast and Ovarian Cancer Susceptibility Gene BRCA1. Science.

[58] Kandoth C., McLellan M.D., Vandin F., Ye K., Niu B., Lu C., Xie M., Zhang Q., McMichael J.F., Wyczalkowski M.A. (2013). Mutational Landscape and Significance across 12 Major Cancer Types. Nature.

[59] Guccini I., Serio D., Condò I., Rufini A., Tomassini B., Mangiola A., Maira G., Anile C., Fina D., Pallone F. (2011). Frataxin Participates to the Hypoxia-Induced Response in Tumors. Cell Death Dis..

[60] Filippakopoulos P., Knapp S. (2014). Targeting Bromodomains: Epigenetic Readers of Lysine Acetylation. Nat. Rev. Drug Discov..

[61] Ruvolo P.P. (2019). Role of Protein Phosphatases in the Cancer Microenvironment. Biochim. Biophys. Acta Mol. Cell Res..

[62] Sauer S. (2015). Ligands for the Nuclear Peroxisome Proliferator-Activated Receptor Gamma. Trends Pharmacol. Sci..

[63] Savojardo C., Manfredi M., Martelli P.L., Casadio R. (2021). Solvent Accessibility of Residues Undergoing Pathogenic Variations in Humans: From Protein Structures to Protein Sequences. Front. Mol. Biosci..

[64] Gilis D., Rooman M. (1996). Stability Changes upon Mutation of Solvent-Accessible Residues in Proteins Evaluated by Database-Derived Potentials. J. Mol. Biol..

[65] Wei Q., Xu Q., Dunbrack R.L. (2013). Prediction of Phenotypes of Missense Mutations in Human Proteins from Biological Assemblies. Proteins.

[66] Duning K., Wennmann D.O., Bokemeyer A., Reissner C., Wersching H., Thomas C., Buschert J., Guske K., Franzke V., Flöel A. (2013). Common Exonic Missense Variants in the C2 Domain of the Human KIBRA Protein Modify Lipid Binding and Cognitive Performance. Transl. Psychiatry.

[67] Feinberg H., Rowntree T.J.W., Tan S.L.W., Drickamer K., Weis W.I., Taylor M.E. (2013). Common Polymorphisms in Human Langerin Change Specificity for Glycan Ligands. J. Biol. Chem..

[68] Haraksingh R.R., Snyder M.P. (2013). Impacts of Variation in the Human Genome on Gene Regulation. J. Mol. Biol..

[69] Silva J.L., De Moura Gallo C.V., Costa D.C.F., Rangel L.P. (2014). Prion-like Aggregation of Mutant P53 in Cancer. Trends Biochem. Sci..

[70] Capriotti E., Fariselli P., Casadio R. (2005). I-Mutant2.0: Predicting Stability Changes upon Mutation from the Protein Sequence or Structure. Nucleic Acids Res..

[71] Zhang Z., Wang L., Gao Y., Zhang J., Zhenirovskyy M., Alexov E. (2012). Predicting Folding Free Energy Changes upon Single Point Mutations. Bioinforma. Oxf. Engl..

[72] Wang Z., Moult J. (2001). SNPs, Protein Structure, and Disease. Hum. Mutat..

[73] Yue P., Li Z., Moult J. (2005). Loss of Protein Structure Stability as a Major Causative Factor in Monogenic Disease. J. Mol. Biol..

[74] Martelli P.L., Fariselli P., Savojardo C., Babbi G., Aggazio F., Casadio R. (2016). Large Scale Analysis of Protein Stability in OMIM Disease Related Human Protein Variants. BMC Genom..

[75] Soragni A., Janzen D.M., Johnson L.M., Lindgren A.G., Thai-Quynh Nguyen A., Tiourin E., Soriaga A.B., Lu J., Jiang L., Faull K.F. (2016). A Designed Inhibitor of P53 Aggregation Rescues P53 Tumor Suppression in Ovarian Carcinomas. Cancer Cell.

[76] Yue P., Moult J. (2006). Identification and Analysis of Deleterious Human SNPs. J. Mol. Biol..

[77] Pasquo A., Consalvi V., Knapp S., Alfano I., Ardini M., Stefanini S., Chiaraluce R. (2012). Structural Stability of Human Protein Tyrosine Phosphatase ρ Catalytic Domain: Effect of Point Mutations. PLoS ONE.

[78] Grothe H.L., Little M.R., Sjogren P.P., Chang A.A., Nelson E.F., Yuan C. (2013). Altered Protein Conformation and Lower Stability of the Dystrophic Transforming Growth Factor Beta-Induced Protein Mutants. Mol. Vis..

[79] Khan S., Vihinen M. (2010). Performance of Protein Stability Predictors. Hum. Mutat..

[80] Waters P.J. (2001). Degradation of Mutant Proteins, Underlying “Loss of Function” Phenotypes, Plays a Major Role in Genetic Disease. Curr. Issues Mol. Biol..

[81] Gummlich L. (2021). ATO Stabilizes Structural P53 Mutants. Nat. Rev. Cancer.

[82] Bullock A.N., Henckel J., Fersht A.R. (2000). Quantitative Analysis of Residual Folding and DNA Binding in Mutant P53 Core Domain: Definition of Mutant States for Rescue in Cancer Therapy. Oncogene.

[83] Williams R.S., Chasman D.I., Hau D.D., Hui B., Lau A.Y., Glover J.N.M. (2003). Detection of Protein Folding Defects Caused by BRCA1-BRCT Truncation and Missense Mutations. J. Biol. Chem..

[84] Rowling P.J.E., Cook R., Itzhaki L.S. (2010). Toward Classification of BRCA1 Missense Variants Using a Biophysical Approach. J. Biol. Chem..

[85] Petrosino M., Pasquo A., Novak L., Toto A., Gianni S., Mantuano E., Veneziano L., Minicozzi V., Pastore A., Puglisi R. (2019). Characterization of Human Frataxin Missense Variants in Cancer Tissues. Hum. Mutat..

[86] Lori C., Lantella A., Pasquo A., Alexander L.T., Knapp S., Chiaraluce R., Consalvi V. (2013). Effect of Single Amino Acid Substitution Observed in Cancer on Pim-1 Kinase Thermodynamic Stability and Structure. PLoS ONE.

[87] Fiorillo A., Petrosino M., Ilari A., Pasquo A., Cipollone A., Maggi M., Chiaraluce R., Consalvi V. (2018). The Phosphoglycerate Kinase 1 Variants Found in Carcinoma Cells Display Different Catalytic Activity and Conformational Stability Compared to the Native Enzyme. PLoS ONE.

[88] Williams R.S., Glover J.N.M. (2003). Structural Consequences of a Cancer-Causing BRCA1-BRCT Missense Mutation. J. Biol. Chem..

[89] Venkitaraman A.R. (2002). Cancer Susceptibility and the Functions of BRCA1 and BRCA2. Cell.

[90] Yarden R.I., Brody L.C. (1999). BRCA1 Interacts with Components of the Histone Deacetylase Complex. Proc. Natl. Acad. Sci. USA.

[91] Cantor S.B., Bell D.W., Ganesan S., Kass E.M., Drapkin R., Grossman S., Wahrer D.C., Sgroi D.C., Lane W.S., Haber D.A. (2001). BACH1, a Novel Helicase-like Protein, Interacts Directly with BRCA1 and Contributes to Its DNA Repair Function. Cell.

[92] Yu X., Wu L.C., Bowcock A.M., Aronheim A., Baer R. (1998). The C-Terminal (BRCT) Domains of BRCA1 Interact in Vivo with CtIP, a Protein Implicated in the CtBP Pathway of Transcriptional Repression. J. Biol. Chem..

[93] Li S., Chen P.L., Subramanian T., Chinnadurai G., Tomlinson G., Osborne C.K., Sharp Z.D., Lee W.H. (1999). Binding of CtIP to the BRCT Repeats of BRCA1 Involved in the Transcription Regulation of P21 Is Disrupted upon DNA Damage. J. Biol. Chem..

[94] Schuster-Böckler B., Bateman A. (2008). Protein Interactions in Human Genetic Diseases. Genome Biol..

[95] Teng S., Madej T., Panchenko A., Alexov E. (2009). Modeling Effects of Human Single Nucleotide Polymorphisms on Protein-Protein Interactions. Biophys. J..

[96] David A., Sternberg M.J.E. (2015). The Contribution of Missense Mutations in Core and Rim Residues of Protein-Protein Interfaces to Human Disease. J. Mol. Biol..

[97] Dixit A., Verkhivker G.M. (2009). Hierarchical Modeling of Activation Mechanisms in the ABL and EGFR Kinase Domains: Thermodynamic and Mechanistic Catalysts of Kinase Activation by Cancer Mutations. PLoS Comput. Biol..

[98] Dixit A., Yi L., Gowthaman R., Torkamani A., Schork N.J., Verkhivker G.M. (2009). Sequence and Structure Signatures of Cancer Mutation Hotspots in Protein Kinases. PLoS ONE.

[99] Dixit A., Torkamani A., Schork N.J., Verkhivker G. (2009). Computational Modeling of Structurally Conserved Cancer Mutations in the RET and MET Kinases: The Impact on Protein Structure, Dynamics, and Stability. Biophys. J..

[100] Acuner Ozbabacan S.E., Gursoy A., Keskin O., Nussinov R. (2010). Conformational Ensembles, Signal Transduction and Residue Hot Spots: Application to Drug Discovery. Curr. Opin. Drug Discov. Devel..

[101] Bauer-Mehren A., Furlong L.I., Rautschka M., Sanz F. (2009). From SNPs to Pathways: Integration of Functional Effect of Sequence Variations on Models of Cell Signalling Pathways. BMC Bioinform..

[102] Vanunu O., Magger O., Ruppin E., Shlomi T., Sharan R. (2010). Associating Genes and Protein Complexes with Disease via Network Propagation. PLoS Comput. Biol..

[103] Barabasi A.L., Gulbahce N., Loscalzo J. (2011). Network Medicine: A Network-Based Approach to Human Disease. Nat. Rev. Genet..

[104] Akhavan S., Miteva M.A., Villoutreix B.O., Venisse L., Peyvandi F., Mannucci P.M., Guillin M.C., Bezeaud A. (2005). A Critical Role for Gly25 in the B Chain of Human Thrombin. J. Thromb. Haemost. JTH.

[105] Gallione C., Aylsworth A.S., Beis J., Berk T., Bernhardt B., Clark R.D., Clericuzio C., Danesino C., Drautz J., Fahl J. (2010). Overlapping Spectra of SMAD4 Mutations in Juvenile Polyposis (JP) and JP-HHT Syndrome. Am. J. Med. Genet. A.

[106] Sayed M.G., Ahmed A.F., Ringold J.R., Anderson M.E., Bair J.L., Mitros F.A., Lynch H.T., Tinley S.T., Petersen G.M., Giardiello F.M. (2002). Germline SMAD4 or BMPR1A Mutations and Phenotype of Juvenile Polyposis. Ann. Surg. Oncol..

[107] Jung B., Staudacher J.J., Beauchamp D. (2017). Transforming Growth Factor β Superfamily Signaling in Development of Colorectal Cancer. Gastroenterology.

[108] Massagué J. (2008). TGFbeta in Cancer. Cell.

[109] Lori L., Pasquo A., Lori C., Petrosino M., Chiaraluce R., Tallant C., Knapp S., Consalvi V. (2016). Effect of BET Missense Mutations on Bromodomain Function, Inhibitor Binding and Stability. PLoS ONE.

[110] Stevanin G., Hahn V., Lohmann E., Bouslam N., Gouttard M., Soumphonphakdy C., Welter M.-L., Ollagnon-Roman E., Lemainque A., Ruberg M. (2004). Mutation in the Catalytic Domain of Protein Kinase C Gamma and Extension of the Phenotype Associated with Spinocerebellar Ataxia Type 14. Arch. Neurol..

[111] Dehal P., Satou Y., Campbell R.K., Chapman J., Degnan B., De Tomaso A., Davidson B., Di Gregorio A., Gelpke M., Goodstein D.M. (2002). The Draft Genome of Ciona Intestinalis: Insights into Chordate and Vertebrate Origins. Science.

[112] Takamiya O., Seta M., Tanaka K., Ishida F. (2002). Human Factor VII Deficiency Caused by S339C Mutation Located Adjacent to the Specificity Pocket of the Catalytic Domain. Clin. Lab. Haematol..

[113] Zhang Z., Teng S., Wang L., Schwartz C.E., Alexov E. (2010). Computational Analysis of Missense Mutations Causing Snyder-Robinson Syndrome. Hum. Mutat..

[114] Zhang Z., Norris J., Schwartz C., Alexov E. (2011). In Silico and in Vitro Investigations of the Mutability of Disease-Causing Missense Mutation Sites in Spermine Synthase. PLoS ONE.

[115] Wang Z., Shen D., Parsons D.W., Bardelli A., Sager J., Szabo S., Ptak J., Silliman N., Peters B.A., van der Heijden M.S. (2004). Mutational Analysis of the Tyrosine Phosphatome in Colorectal Cancers. Science.

[116] Compiani M., Capriotti E. (2013). Computational and Theoretical Methods for Protein Folding. Biochemistry.

[117] Marabotti A., Scafuri B., Facchiano A. (2020). Predicting the Stability of Mutant Proteins by Computational Approaches: An Overview. Brief. Bioinform..

[118] Kumar M.D., Bava K.A., Gromiha M.M., Prabakaran P., Kitajima K., Uedaira H., Sarai A. (2006). ProTherm and ProNIT: Thermodynamic Databases for Proteins and Protein-Nucleic Acid Interactions. Nucleic Acids Res..

[119] Fernald G.H., Capriotti E., Daneshjou R., Karczewski K.J., Altman R.B. (2011). Bioinformatics Challenges for Personalized Medicine. Bioinformatics.

[120] Niroula A., Vihinen M. (2016). Variation Interpretation Predictors: Principles, Types, Performance, and Choice. Hum. Mutat..

[121] Capriotti E., Ozturk K., Carter H. (2019). Integrating Molecular Networks with Genetic Variant Interpretation for Precision Medicine. Wiley Interdiscip. Rev. Syst. Biol. Med..

[122] Capriotti E., Calabrese R., Casadio R. (2006). Predicting the Insurgence of Human Genetic Diseases Associated to Single Point Protein Mutations with Support Vector Machines and Evolutionary Information. Bioinformatics.

[123] Thomas P.D., Kejariwal A. (2004). Coding Single-Nucleotide Polymorphisms Associated with Complex vs. Mendelian Disease: Evolutionary Evidence for Differences in Molecular Effects. Proc. Natl. Acad. Sci. USA.

[124] Sim N.-L., Kumar P., Hu J., Henikoff S., Schneider G., Ng P.C. (2012). SIFT Web Server: Predicting Effects of Amino Acid Substitutions on Proteins. Nucleic Acids Res..

[125] Bromberg Y., Rost B. (2007). SNAP: Predict Effect of Non-Synonymous Polymorphisms on Function. Nucleic Acids Res..

[126] Kabsch W., Sander C. (1983). Dictionary of Protein Secondary Structure: Pattern Recognition of Hydrogen-Bonded and Geometrical Features. Biopolymers.

[127] Baugh E.H., Ke H., Levine A.J., Bonneau R.A., Chan C.S. (2018). Why Are There Hotspot Mutations in the TP53 Gene in Human Cancers?. Cell Death Differ..

[128] Kim M.P., Lozano G. (2018). Mutant P53 Partners in Crime. Cell Death Differ..

[129] Bloom J.D., Labthavikul S.T., Otey C.R., Arnold F.H. (2006). Protein Stability Promotes Evolvability. Proc. Natl. Acad. Sci. USA.

[130] Tokuriki N., Tawfik D.S. (2009). Stability Effects of Mutations and Protein Evolvability. Curr. Opin. Struct. Biol..

[131] Vihinen M. (2021). Functional Effects of Protein Variants. Biochimie.

[132] Iqbal S., Pérez-Palma E., Jespersen J.B., May P., Hoksza D., Heyne H.O., Ahmed S.S., Rifat Z.T., Rahman M.S., Lage K. (2020). Comprehensive Characterization of Amino Acid Positions in Protein Structures Reveals Molecular Effect of Missense Variants. Proc. Natl. Acad. Sci. USA.

[133] Andreoletti G., Pal L.R., Moult J., Brenner S.E. (2019). Reports from the Fifth Edition of CAGI: The Critical Assessment of Genome Interpretation. Hum. Mutat..

[134] Savojardo C., Petrosino M., Babbi G., Bovo S., Corbi-Verge C., Casadio R., Fariselli P., Folkman L., Garg A., Karimi M. (2019). Evaluating the Predictions of the Protein Stability Change upon Single Amino Acid Substitutions for the FXN CAGI5 Challenge. Hum. Mutat..

[135] Chandonia J.-M., Adhikari A., Carraro M., Chhibber A., Cutting G.R., Fu Y., Gasparini A., Jones D.T., Kramer A., Kundu K. (2017). Lessons from the CAGI-4 Hopkins Clinical Panel Challenge. Hum. Mutat..

[136] Pal L.R., Kundu K., Yin Y., Moult J. (2017). CAGI4 SickKids Clinical Genomes Challenge: A Pipeline for Identifying Pathogenic Variants. Hum. Mutat..

[137] Stein Y., Aloni-Grinstein R., Rotter V. (2020). Mutant P53 Oncogenicity: Dominant-Negative or Gain-of-Function?. Carcinogenesis.

[138] Uversky V.N. (2016). P53 Proteoforms and Intrinsic Disorder: An Illustration of the Protein Structure–Function Continuum Concept. Int. J. Mol. Sci..

[139] Ribeiro R.C., Sandrini F., Figueiredo B., Zambetti G.P., Michalkiewicz E., Lafferty A.R., DeLacerda L., Rabin M., Cadwell C., Sampaio G. (2001). An Inherited P53 Mutation That Contributes in a Tissue-Specific Manner to Pediatric Adrenal Cortical Carcinoma. Proc. Natl. Acad. Sci. USA.

[140] Latronico A.C., Pinto E.M., Domenice S., Fragoso M.C., Martin R.M., Zerbini M.C., Lucon A.M., Mendonca B.B. (2001). An Inherited Mutation Outside the Highly Conserved DNA-Binding Domain of the P53 Tumor Suppressor Protein in Children and Adults with Sporadic Adrenocortical Tumors. J. Clin. Endocrinol. Metab..

[141] Bhattacharya R., Rose P.W., Burley S.K., Prlić A. (2017). Impact of Genetic Variation on Three Dimensional Structure and Function of Proteins. PLoS ONE.

[142] Williams M.J., Sottoriva A., Graham T.A. (2019). Measuring Clonal Evolution in Cancer with Genomics. Annu. Rev. Genomics Hum. Genet..

