# Supplementary material for: Analysis and Interpretation of the Impact of Missense Variants in Cancer
Source: Int J Mol Sci. 2021 May 21;22(11):5416. doi: 10.3390/ijms22115416 (PMC8196604; doi:10.3390/ijms22115416)
Supplement: Supplementary file 1 [file ijms-22-05416-s001.zip › 20210503-ijms-supmat.pdf]

SUPPLEMENTARY MATERIALS

Analysis and interpretation of the impact of missense variants in cancer

Maria Petrosino[1], Leonore Novak[1], Alessandra Pasquo[2], Roberta Chiaraluce[1], Paola Turina[3], Emidio Capriotti[3], Valerio Consalvi[1]**

[1] Dipartimento Scienze Biochimiche "A. Rossi Fanelli", Sapienza University of Rome, Roma (Italy)
[2] ENEA CR Frascati, Diagnostics and Metrology Laboratory FSN-TECFIS-DIM Frascati RM (Italy)
[3] Dipartimento di Farmacia e Biotecnologie (FaBiT), University of Bologna, Bologna (Italy)

Contacts: Emidio Capriotti (emidio.capriotti@unibo.it)
Valerio Consalvi (valerio.consalvi@uniroma1.it)

1 Dataset composition

The dataset used in our analysis consists of 164 variants from 11 proteins from genes annotated as *Tier 1* in the Cancer Census Gene list of the COSMIC database (BRCA1, BRD3, BRD4, PIM-1, PPARγ, PTPρ, p16, p53), or from other cancer-related genes (BRD2, hFXN, PGK1). Among the latter group of genes, BRD2 shares more than 50% identity with the *Tier*1 gene BRD3, hFXN has been implicated in mice tumorigenesis (Thierbach *et al.*, 2005, 2012), and PGK1 is reported as a mice candidate cancer causing gene in the CCDG database (Abbott *et al.*, 2015). The dataset was collected after extensive literature and databases search for cancer-related variants, for which the change in folding free energy difference upon mutation ($\Delta\Delta G_f$) had been experimentally determined. When available, for each variant the melting temperature change (ΔT_m) was also collected. The $\Delta\Delta G_f$ and ΔT_mvalues for the variants in the p53 C-terminal domain, which were measured in its tetrameric state, were divided by 4. Our dataset is composed of variants which are either detected in normal and tumor tissues, or engineered.

The mutations are classified as highly destabilizing ($\Delta\Delta G_f$>2.0 kcal/mol) or not ($\Delta\Delta G_f$≤2.0 kcal/mol). A second classification is based on the annotation retrieved in the Cancer Mutation Census from COSMIC (Tate et al., 2019). According to such annotation, variants were classified as *cancer-causing* (those annotated as *Tiers 1,2,3*) or *benign* (those annotated as *"Other").* Among the 164 variants collected, only 97 have been annotated in the Cancer Mutation Census database. For a better assessment of the performance of Meta-SNP we also considered a subset of 82 mutants obtained removing 15 mutants included in the Meta-SNP training set. A summary of the composition of our database is reported in Table S1.

2 Variant effect predictions and classification

The impact of the selected variants on protein stability was predicted using FoldX (Schymkowitz *et al.*, 2005), which returns in output the $\Delta\Delta G_f$ of folding. In our analysis, we generated 10 possible models of the mutated structure and calculated the average $\Delta\Delta G_f$ value on the 10 models.

The pathogenicity of each variant was predicted using Meta-SNP (Capriotti *et al.*, 2013), a binary classifier combining the prediction outputs of 4 state-of-the-art methods, namely PhD-SNP (Capriotti *et al.*, 2006), PANTHER (Thomas and Kejariwal, 2004), SIFT (Sim *et al.*, 2012) and SNAP (Bromberg and Rost, 2007). The output of the method is a probabilistic score ranging from 0 to 1.

The binary classification tasks for both FoldX and Meta-SNP were performed by optimizing the thresholds that better discriminated between highly destabilizing ($\Delta\Delta G_f$>2.0 kcal/mol) and not destabilizing ($\Delta\Delta G_f$≤2.0 kcal/mol) variants, or between *cancer-causing* (*Tier 1-3*) and *benign* (*'Other'*) variants.

3 Variant classification features

In our analysis, the solvent accessibility of the mutated sites was calculated by using the DSSP program (Kabsch and Sander, 1983). The Relative Solvent Accessibility (RSA) of each amino acid was obtained by normalizing its solvent accessibility with the maximum solvent accessibility of the same amino acid. In this work we used the estimation of the maximum accessibility of the amino acids calculated by Rost and Sander (Rost and Sander, 1994).

The conservation score of the wild-type residue (f_{WT}) is provided in the output of the Meta-SNP server, which calculates a multiple sequence alignment of the homolog proteins retrieved by the BLAST algorithm (Altschul et al., 1997) on the UniRef90 database (Suzek et al., 2007), as suggested in the PhD-SNP article (Capriotti et al., 2006). The f_{WT} is the fraction of the wild-type residue among all the aligned sequences in the mutated site.

4 Measures of performance

Performance in regression mode

For evaluating the performance of FoldX in the regression task, we compared the predicted and experimental values of the variation of folding free energy change ($\Delta\Delta G_f$) upon amino acid substitution. The standard scoring values calculated in our assessment are the Pearson, Spearman and Kendall-Tau correlation coefficients (r_P, r_S, *and* r_{KT} respectively), the root mean square error (RMSE) and the mean absolute error (MAE). They are defined as follows:

$$r_P = \frac{n\sum_{i=1}^{n} y_i \bar{y}_i - \left(\sum_{i=1}^{n} y_i\right)\left(\sum_{i=1}^{n} \bar{y}_i\right)}{\sqrt{\left[n\sum_{i=1}^{n} y_i^2 - \left(\sum_{i=1}^{n} y_i\right)^2\right]}\sqrt{\left[n\sum_{i=1}^{n} \bar{y}_i^2 - \left(\sum_{i=1}^{n} \bar{y}_i\right)^2\right]}} \quad [1]$$

$$r_S = \frac{n\sum_{i=1}^{n} r(y_i) r(\bar{y}_i) - \left(\sum_{i=1}^{n} r(y_i)\right)\left(\sum_{i=1}^{n} r(\bar{y}_i)\right)}{\sqrt{\left[n\sum_{i=1}^{n} r(y_i)^2 - \left(\sum_{i=1}^{n} r(y_i)\right)^2\right]}\sqrt{\left[n\sum_{i=1}^{n} r(\bar{y}_i)^2 - \left(\sum_{i=1}^{n} r(\bar{y}_i)\right)^2\right]}} \quad [2]$$

$$r_{KT} = \frac{2}{n(n-1)} = \sum_{i=1}^{n}\sum_{j=1}^{i-1} sign(y_i - y_j)\ sign(\bar{y}_i - \bar{y}_j) \quad [3]$$

$$RMSE = \sqrt{\frac{\sum_{i=1}^{n}(y_i - \bar{y}_i)^2}{n}} \quad [4]$$

$$MAE = \frac{\sum_{i=1}^{n}\left|y_i - \bar{y}_i\right|}{n} \quad [5]$$

where y_i and $\bar{y}_i$ and are the experimental and predicted $\Delta\Delta G_f$ values, respectively, $r(y_i)$ and $r(\bar{y}_i)$ their ranks.

Performance of the binary classifiers

In our analysis, we assessed the performance of FoldX and Meta-SNP as binary classifiers for predicting highly destabilizing variants ($\Delta\Delta G_f$>2.0 kcal/mol) and *Tier 1-3* variants from the CMC database. In all the performance measures, we labelled as positive the highly destabilizing or the *Tier 1-3* variants, and as negative those variants with $\Delta\Delta G_f$≤2.0 kcal/mol or annotated as *"Other"*. The predictors performances were evaluated using the following metrics:

$$PPV = \frac{TP}{TP+FP} \quad TPR = \frac{TP}{TP+FN}$$

$$NPV = \frac{TN}{TN+FN} \quad TNR = \frac{TN}{TN+FP} \quad [6]$$

$$F1 = \frac{2TP}{2TP+FP+FN} \quad Q_2 = \frac{TP+TN}{TP+FP+TN+FN}$$

where *TN* and *FN* are the true and false negative, *TP* and *FP* are the true and false positive, *PPV* and *NPV* are the positive and negative predicted values, and *TPR* and *TNR* are true positive and negative rates. The *F1* score is the harmonic mean between *PPV* and *TPR*, and Q_2 is the overall accuracy.

The Matthew's correlation coefficient *MCC* was computed as:

$$MCC = \frac{TP \times TN - FP \times FN}{\sqrt{(TP+FP)\ (TP+FN)\ (TN+FP)\ (TN+FN)}} \qquad [7]$$

The Area Under the Receiver Operating Characteristic (ROC) Curve (AUC) was calculated by plotting the True Positive Rate (*TPR*) as a function of the False Positive Rate (*FPR*=1-*TNR*) at different classification thresholds.

For a better generalization of the threshold optimization process in the prediction of high destabilizing and putative cancer-driving variants, we estimated the performance of the methods using a 5-fold cross-validation process. The reported optimal thresholds and the measures of the performance represent the average values obtained on the 5 subsets.

Supplementary Tables

Protein	PDB	Chain	$\Delta\Delta G_f$>2.0 kcal/mol	$\Delta\Delta G_f$≤2.0 kcal/mol	$N_{\Delta\Delta G}$	CMC *Tier 1-3*	CMC *'Other'*	N_{CMC}
ALL	–	–	53	111	164	24	73	97
p53	3SAK	A	13	45	58	3	27	30
p53	2OCJ	A	15	27	42	15	17	32
BRCA	1JNX	X	9	15	24	4	6	10
hFXN	1EKG	A	4	3	7	0	7	7
PGK1	2XE7	A	3	4	7	0	0	0
PPARγ	1PRG	A	0	6	6	1	1	2
BRD2(1)	4ALG	A	5	0	5	0	4	4
PIM-1	1XWS	A	4	0	4	0	4	4
p16	1DC2	A	0	3	3	1	2	3
PTPρ	2OOQ	A	0	3	3	0	0	0
BRD2(2)	3ONI	A	0	2	2	0	2	2
BRD3(2)	3S92	A	0	1	1	0	1	1
BRD4(1)	3MXF	A	0	1	1	0	1	1
BRD4(2)	2OUO	A	0	1	1	0	1	1

Table S1. Composition of the dataset according to the number of variants in each protein and class. In the training set of Meta-SNP there were present 10 mutations from p53, 3 mutations from BRCA1 and 2 mutations from PIM1.

Protein	r_P	*p-value*$_P$	RMSE	MAE	r_S	*p-value*$_S$	r_{KT}	*p-value*$_{KT}$	N
ALL	0.502	7.20E-12	2.09	1.39	0.543	6.20E-14	0.372	1.70E-12	164
ALL*	0.558	9.90E-15	1.82	1.32	0.542	8.30E-14	0.373	1.80E-12	163
p53 (3SAK)	0.579	1.90E-06	1.20	0.97	0.572	2.80E-06	0.380	2.70E-05	58
p53 (2OCJ)	0.756	7.20E-09	1.78	1.26	0.710	1.40E-07	0.490	4.80E-06	42
BRCA1	0.465	2.20E-02	3.10	1.65	0.723	6.70E-05	0.500	4.20E-04	24
BRCA1*	0.739	5.70E-05	1.52	1.14	0.733	6.90E-05	0.526	2.80E-04	23
BRD	0.176	6.30E-01	3.04	2.64	0.309	3.90E-01	0.244	3.80E-01	10
hFXN	0.651	1.10E-01	1.64	1.31	0.429	3.40E-01	0.333	3.80E-01	7
PGK1	0.791	3.40E-02	1.44	1.19	0.500	2.50E-01	0.333	3.80E-01	7
PPARγ	0.475	3.40E-01	2.84	1.70	0.429	4.00E-01	0.200	7.20E-01	6
PIM-1	-0.603	4.00E-01	3.51	3.24	-0.200	8.00E-01	0.000	1.30E+00	4
PTPρ	0.813	4.00E-01	2.40	1.76	0.500	6.70E-01	0.333	1.00E+00	3
p16	-0.570	6.10E-01	3.09	2.43	-1.000	0.00E+00	-1.000	3.30E-01	3

Table S2. Performance of FoldX in the prediction of the ΔΔGf values. The Pearson, Spearman and Kendall-Tau correlation coefficients (r_P, r_S and r_{KT}), as well as the Root Mean Square Error (RMSE) and the Mean Absolute Error (MAE), expressed in kcal/mol. are defined in Supplementary Materials. *Fitting after removing the outlier variant BRCA1 p.G1788V.

Protein	TH	Q2	TNR	NPV	TPR	PPV	MCC	F1	AUC	N_{HD}/N_T
ALL	1.23	0.774	0.757	0.895	0.824	0.631	0.552	0.705	0.847	53/164
p53 (3SAK)	1.30	0.741	0.736	0.917	0.816	0.455	0.445	0.558	0.832	13/58
p53 (2OCJ)	2.31	0.905	0.917	0.946	0.810	0.908	0.781	0.821	0.948	15/42
BRCA1*	1.23	0.833	0.800	0.923	0.889	0.727	0.669	0.800	0.904	9/24

Table S3. Performance of FoldX in the prediction of highly destabilizing variants. Highly destabilizing variants have $\Delta\Delta G_f$>2.0 kcal/mol. The measures of performance are defined in the Supplementary Materials. TH is the optimized threshold for maximizing both TNR and TPR. N_{HD} and N_T represent the highly destabilizing and total number of variants, respectively. The optimization process was performed using a 5-fold cross-validation procedure. The measures of performance represent the average values on the 5 subsets. *For BRCA1 the performance was calculated using the optimization threshold obtained on the full set because the number of mutations is too small.

Prediction task	TH	Q2	TNR	NPV	TPR	PPV	MCC	F1	AUC	N_P/N_T
Meta-SNP (ΔΔG>2)	0.66	0.732	0.763	0.832	0.646	0.565	0.402	0.595	0.737	53/164
Meta-SNP§ (Tier 1-3)	0.71	0.768	0.787	0.931	0.747	0.342	0.370	0.437	0.781	12/82
FoldX (*Tier 1-3*)	2.69	0.732	0.818	0.833	0.493	0.533	0.333	0.471	0.733	24/97

Table S4. Performance of FoldX and Meta-SNP in the prediction of highly destabilizing and causative variants. Highly destabilizing variants have $\Delta\Delta G_f$>2.0 kcal/mol. Causative variants are annotated as *Tier 1-3* in the COSMIC database. The measures of performance are defined in the Supplementary Materials. TH is the optimized threshold for maximizing both TNR and TPR. N_P and N_T represent the positive (P) and total (T) number of variants, respectively. The Positive variants are either highly destabilizing ($\Delta\Delta G_f$>2.0 kcal/mol) or *Tier 1-3* variants. § Performance calculated removing 15 mutations included in the training set of Meta-SNP. The optimization process was performed using a 5-fold cross-validation procedure. The measures of performance represent the average values on the 5 subsets.

Supplementary Figure

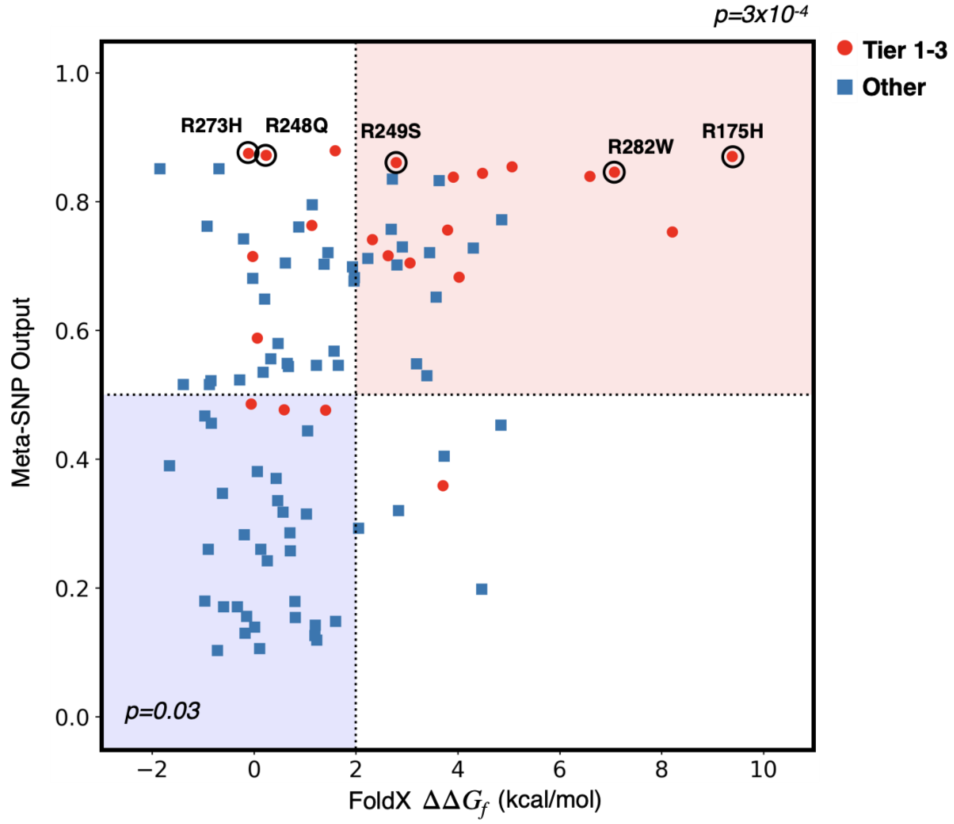

Fig. S1. Enrichment in *Tier 1-3* variants in the subset of mutants with predicted $\Delta\Delta G_f$ > 2.0 kcal/mol and Meta-SNP output >0.5 (light red). The opposite region (light blue) is depleted of *Tier* 1-3 variants. P-values are calculated by using a binomial distribution with a success probability of 0.247. Five hotspot mutants are highlighted with black circles.

Supplementary File 1

Dataset of 164 mutations in 11 proteins. For each mutation, the following information is reported: The protein name (PROT), the sequence mutant (SMUT), the protein structure identifier (PDB), the structure mutation (MUT), the relative solvent accessibility (RSA), the frequency of the wild-type residue in the protein sequence profile (FWT), the predicted $\Delta\Delta G_f$ by FoldX (FOLDX), its standard deviation (STDEV), the output of Meta-SNP (METASNP), the experimental $\Delta\Delta G_f$ and ΔT_m, the annotation on the Cancer Mutation Census (CMC), the number of mutated tissue samples (CMS), the publication identifier reporting the experimental $\Delta\Delta G_f$ and ΔT_m (PMID) and the tumor tissues in which the mutations are detected (TISSUE). The experimental $\Delta\Delta G_f$ and ΔT_m values for the p53 C-terminal tetramerization domain (3SAK) were divided by 4.

References

Abbott,K.L. *et al.* (2015) The Candidate Cancer Gene Database: a database of cancer driver genes from forward genetic screens in mice. *Nucleic Acids Res.*, **43**, D844-848.

Altschul,S.F. *et al.* (1997) Gapped BLAST and PSI-BLAST: a new generation of protein database search programs. *Nucleic Acids Res*, **25**, 3389–402.

Bromberg,Y. and Rost,B. (2007) SNAP: predict effect of non-synonymous polymorphisms on function. *Nucleic Acids Res*, **35**, 3823–35.

Capriotti,E. *et al.* (2013) Collective judgment predicts disease-associated single nucleotide variants. *BMC Genomics*, **14 Suppl 3**, S2.

Capriotti,E. *et al.* (2006) Predicting the insurgence of human genetic diseases associated to single point protein mutations with support vector machines and evolutionary information. *Bioinformatics*, **22**, 2729–34.

Kabsch,W. and Sander,C. (1983) Dictionary of protein secondary structure: pattern recognition of hydrogen-bonded and geometrical features. *Biopolymers*, **22**, 2577–2637.

Rost,B. and Sander,C. (1994) Conservation and prediction of solvent accessibility in protein families. *Proteins*, **20**, 216–226.

Schymkowitz,J. *et al.* (2005) The FoldX web server: an online force field. *Nucleic Acids Res*, **33**, W382-8.

Sim,N.-L. *et al.* (2012) SIFT web server: predicting effects of amino acid substitutions on proteins. *Nucleic Acids Res.*, **40**, W452-457.

Suzek,B.E. *et al.* (2007) UniRef: comprehensive and non-redundant UniProt reference clusters. *Bioinformatics*, **23**, 1282–8.

Tate,J.G. *et al.* (2019) COSMIC: the Catalogue Of Somatic Mutations In Cancer. *Nucleic Acids Res.*, **47**, D941–D947.

Thierbach,R. *et al.* (2012) Specific alterations of carbohydrate metabolism are associated with hepatocarcinogenesis in mitochondrially impaired mice. *Hum. Mol. Genet.*, **21**, 656–663.

Thierbach,R. *et al.* (2005) Targeted disruption of hepatic frataxin expression causes impaired mitochondrial function, decreased life span and tumor growth in mice. *Hum. Mol. Genet.*, **14**, 3857–3864.

Thomas,P.D. and Kejariwal,A. (2004) Coding single-nucleotide polymorphisms associated with complex vs. Mendelian disease: evolutionary evidence for differences in molecular effects. *Proc Natl Acad Sci U A*, **101**, 15398–403.
